# Phase-resolved functional lung magnetic resonance imaging for evaluation of lung perfusion and ventilation in fibrosing interstitial lung diseases

**DOI:** 10.1186/s13244-026-02321-5

**Published:** 2026-06-04

**Authors:** Yifei Ni, Hongyi Wang, Anqi Liu, Jianping Wang, Jie Du, Linfeng Xi, Yuhui Qiang, Shiyao Wang, Shi Shu, Jing An, Robert Grimm, Andreas Voskrebenzev, Jens Vogel-Claussen, Ling Zhao, Yanhong Ren, Min Liu

**Affiliations:** 1 Chinese Academy of Medical Sciences & Peking Union Medical College, Beijing, China; 2 Department of Radiology, China–Japan Friendship Hospital, Beijing, China; 3 Department of Pulmonary and Critical Care Medicine, China–Japan Friendship Hospital; National Center for Respiratory Medicine; Institute of Respiratory Medicine, Chinese Academy of Medical Sciences; National Clinical Research Center for Respiratory Diseases, Beijing, China; 4 Capital Medical University, Beijing, China; 5 DL department, Siemens Shenzhen Magnetic Resonance Ltd, Shenzhen, China; 6 Research & Clinical Translation, Magnetic Resonance, Siemens Healthineers AG, Erlangen, Germany; 7 Department of Diagnostic and Interventional Radiology, Charité Universitätsmedizin, Berlin, Germany; 8 Department of Pathology, China–Japan Friendship Hospital, Beijing, China

**Keywords:** Idiopathic pulmonary fibrosis, Interstitial lung disease, Magnetic resonance imaging, Perfusion, Ventilation

## Abstract

**Objective:**

The pathophysiological changes of lung perfusion and ventilation in fibrosing interstitial lung diseases (F-ILD) remain inadequately characterized. This study aimed to analyze lung perfusion and ventilation characteristics in F-ILD patients using phase-resolved functional lung magnetic resonance imaging (PREFUL MRI) as well as their correlation with the severity of F-ILD.

**Materials and methods:**

This cross-sectional study prospectively included 30 patients diagnosed with F-ILD (19 males, 64.6 ± 9.5 years) and 30 age- and sex-matched normal controls. All participants underwent PREFUL MRI as well as pulmonary function tests. High-resolution CT (HRCT) was performed for the patient cohort. Ventilation and perfusion-related parameters obtained from PREFUL MRI were analyzed and correlated with PFTs and fibrotic lesions identified on HRCT.

**Results:**

Compared with normal controls, F-ILD patients showed significant differences in mean perfusion (7.55% vs 4.60%), Mean Ventilation (13.95% vs 18.65%), Perfusion Defect (QDPexclusive) (3.65% vs 15.50%), ventilation-perfusion matched non-defect percentage (VQMnon-defect) (87.55% vs 70.10%), and ventilation-perfusion matched defect percentage (VQMdefect) (0.15% vs 1.35%) (all *p* < 0.05). Mean perfusion correlated positively with DLCO SB (single breath) %pred (ρ = 0.682, *p* < 0.001) and DLCO/VA (alveolar volume) %pred (ρ = 0.634, *p* < 0.001), while QDPexclusive correlated negatively with these parameters. Mean perfusion showed negative correlations with honeycombing, fibrotic lesions, and total interstitial lesion burden on HRCT, whereas QDPexclusive correlated positively with these abnormalities (all *p* < 0.05).

**Conclusion:**

PREFUL MRI provides a quantitative functional evaluation of ventilation and perfusion in F-ILD patients, demonstrating strong correlations with pulmonary function parameters and fibrotic lesions. It shows potential as a valuable monitoring tool enabling severity assessment of F-ILD.

**Critical relevance statement:**

PREFUL MRI provides a non-invasive, free-radiation method in the assessment of ventilation and perfusion in F-ILD, enabling severity evaluation.

**Key Points:**

- In patients with F-ILD, lung perfusion decreased, and ventilation increased.
- Lung ventilation and perfusion correlated with lung function parameters in F-ILD; however, they are similar between idiopathic pulmonary fibrosis (IPF) and other types of F-ILD.
- After controlling demographics, PREFUL MRI perfusion parameters (mean perfusion and QDPexclusive) remain significant, independent predictors of gas-exchange capacity and fibrotic burden

**Graphical Abstract:**

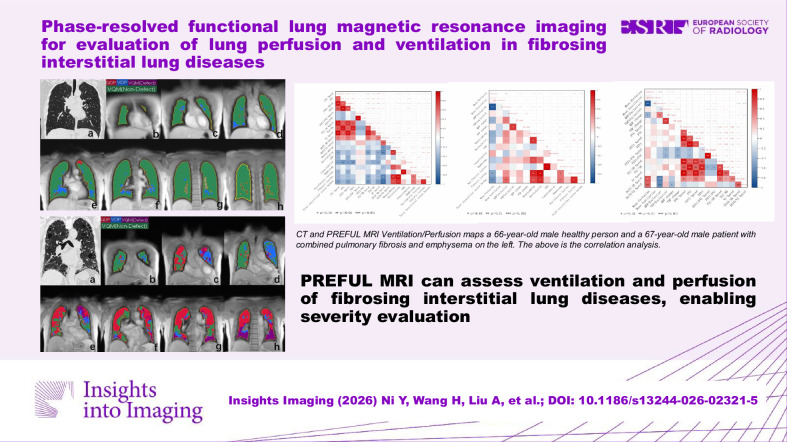

## Introduction

Fibrosing interstitial lung diseases (F-ILD) constitute a clinically significant subgroup marked by progressive pulmonary fibrosis accompanied by persistent inflammatory activity [1, 2]. Idiopathic pulmonary fibrosis (IPF), the prototypical and most aggressive F-ILD variant, demonstrates characteristic radiologic and histopathologic patterns distinct from other fibrosing subtypes [2, 3]. The inexorable progression of these disorders, characterized by fibroblast proliferation, aberrant extracellular matrix deposition, and irreversible architectural distortion of the lung parenchyma, ultimately results in progressive respiratory insufficiency, underscoring the critical need for early diagnostic stratification and longitudinal monitoring to guide therapeutic decision-making.

Current diagnostic paradigms rely principally on high-resolution computed tomography (HRCT) and pulmonary function testing (PFT). HRCT plays a pivotal role in identifying fibrotic patterns and monitoring disease progression, and quantitative CT metrics now enable objective evaluation of fibrosis extent and predictive modeling for disease trajectories; however, frequent use is limited by concerns over radiation exposure. Conventional PFT, though essential for global functional assessment, demonstrates limited sensitivity for detecting regional parenchymal alterations and proves particularly challenging in critically ill patients with compromised respiratory mechanics [4, 5]. Phase-Resolved Functional Lung MRI (PREFUL MRI) is a novel functional imaging technique that evaluates lung perfusion and ventilation by analyzing endogenous proton signal changes during respiratory and circulatory cycles [6]. This radiation-free, free-breathing technique does not require inhaled contrast agents or gadolinium injection and is more readily accepted by patients. PREFUL MRI has been validated for quantitative assessments of perfusion and ventilation in various diseases, such as chronic obstructive pulmonary disease (COPD) [6–11], pulmonary embolism [12–14], asthma [15], and lung transplant patients [16]. Also, it has proven valuable for assessing disease severity and monitoring treatment response [17]. To the best of our knowledge, no studies have yet utilized PREFUL MRI in the assessment of ILD.

The objective of this study was to evaluate lung perfusion and ventilation in F-ILD patients using PREFUL MRI, to investigate its correlation with fibrosis lesions and lung function, and to establish new monitoring and evaluation markers for F-ILD.

## Materials and methods

### Study design and participants

**Fig. 1:**
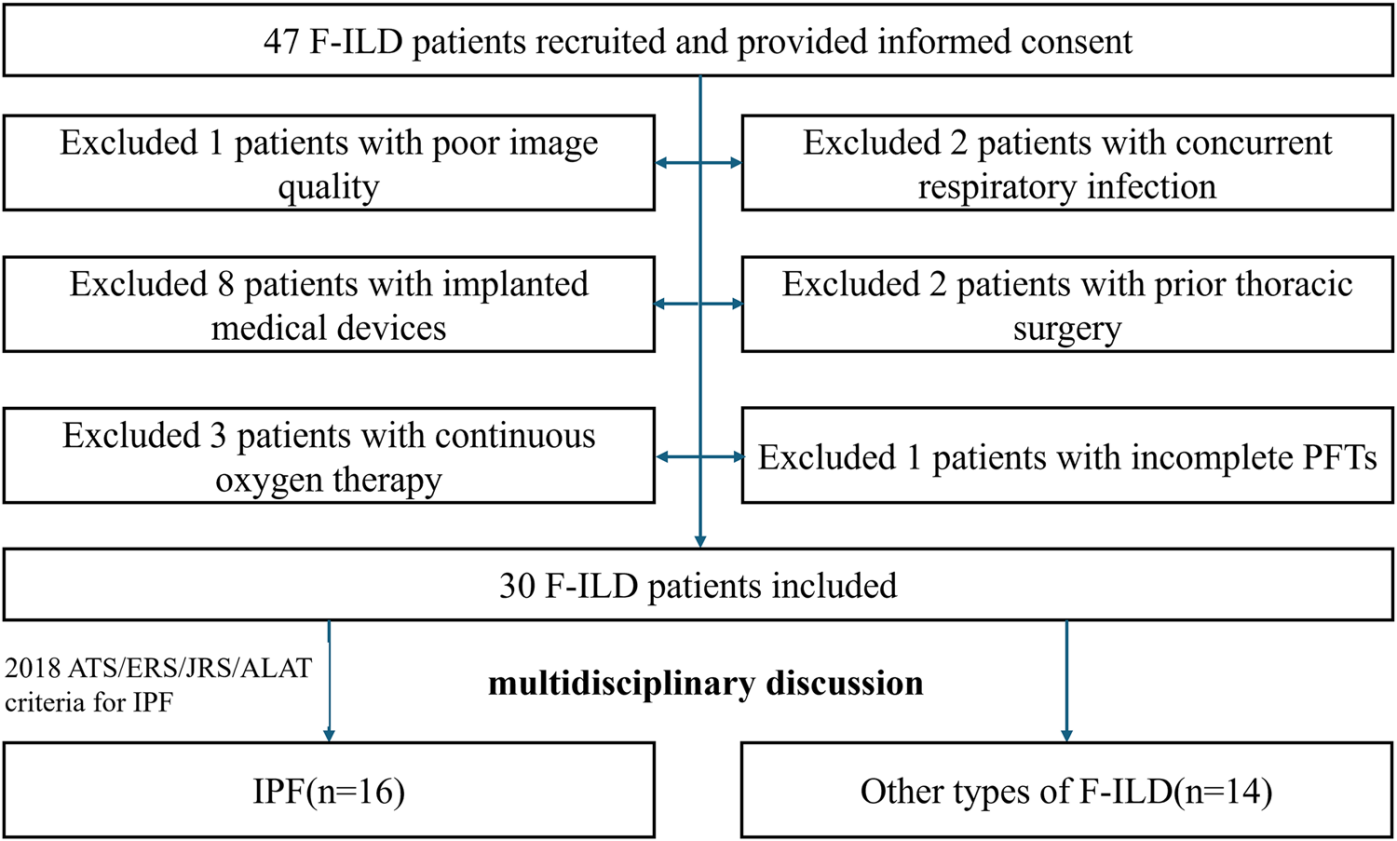
Flowchart of patient inclusion and exclusion criteria. F-ILD, fibrosing interstitial lung diseases; PFTs, pulmonary function tests; IPF, idiopathic pulmonary fibrosis

This prospective cross-sectional study was approved by the institutional ethics committee of China-Japan Friendship Hospital (IRB2024-KY-066), with informed consent from all participants. From January to October 2024, patients who were diagnosed with F-ILD through multidisciplinary discussion were enrolled. Diagnostic criteria for F-ILD [18]: i. Dyspnea at rest/exertion; dry cough /the presence of Velcro crackles; ii. PFTs indicating restrictive ventilatory impairment and diffusion abnormalities; iii. HRCT evidence of fibrotic features, including honeycombing, reticulation, and traction bronchiectasis. Exclusion criteria: i. Presence of implanted medical devices such as stents; ii. Requirement for continuous oxygen therapy or concurrent respiratory infection; iii. Prior thoracic surgery; iv. Inability to complete PREFUL MRI or PFTs. Meanwhile, an age- and sex-matched healthy control group was recruited with inclusion criteria of normal PFTs, Low-dose chest CT (LDCT), and electrocardiography, excluding smokers, individuals with coronary stents/pacemakers, cardiopulmonary surgery history, or thromboembolism. All participants underwent HRCT (for patients)/LDCT (for healthy controls), PFTs, and PREFUL MRI within 48 h. Figure 1 is the flow chart of this study.

### Pulmonary function tests (PFTs)

PFTs were performed using the MasterScreen™ system following ERS/American Thoracic Society guidelines [19, 20]. Spirometric measurements included: forced vital capacity (% predicted; FVC), forced expiratory volume in 1 s (% predicted; FEV₁), FEV₁/FVC ratio (% predicted), vital capacity (% predicted; VC), total lung capacity (% predicted; TLC), and carbon monoxide diffusing capacity (% predicted; DLCO). Percentage of predicted single-breath diffusing capacity for carbon monoxide (DLCO SB %pred), which reflects the overall gas transfer capacity of the lungs. Percentage of predicted diffusing capacity for carbon monoxide normalized to the alveolar volume (DLCO/VA %pred), which provides an index of the efficiency of gas transfer per unit lung volume.

### HRCT scanning protocol

All patients underwent HRCT using multi-detector spiral CT systems (LightSpeed VCT/64 (GE), Aquilion ONE TSX-301C/320, iCT/256, and SOMATOM Definition Flash dual-source CT). Scans were performed in the supine position, arms raised overhead, during full-inspiration breath-hold. The scanning covers the entire lung. The imaging parameters were as follows: tube voltage range of 100–120 kV, tube current range of 100–300 mAs, slice thickness of 0.625–1 mm, table speed of 39.37 mm/s, detector rotation time of 0.5 s, and reconstructed slice thickness of 1–1.25 mm.

### HRCT image analysis

HRCT images in the Digital Imaging and Communications in Medicine format were transferred to a commercial in-house AI workstation (CT quantitative system: SIMBIO-T, Hangzhou Smart Intelligent Technology Co. Ltd) for automatic quantitative analysis of abnormal findings on HRCT. A deep convolutional neural network-based two-dimensional classifier embedded in this AI workstation for diffuse interstitial lung disease (ILD) was used to automatically categorize imaging features into five subtypes: honeycombing, reticulation, emphysema, ground-glass opacity (GGO), and consolidation [21–23]. After lobular segmentation, the program reports the absolute volume (mL) and the percentage of total lung volume occupied by each feature. The whole-lung percentage of fibrotic lesions (%) = % honeycombing + % reticulation. The whole-lung percentage of non-fibrotic lesions (%) = % GGO + % emphysema + % consolidation. Total interstitial lesion burden (%) = % honeycombing + % reticulation + % GGO + % emphysema + % consolidation.

### PREFUL MRI scanning protocol

PREFUL MRI Scanning examinations were performed using a 1.5-T MRI system (MAGNETOM Aera, Siemens Healthineers) in the supine position with 18-channel body and 12-channel spine matrix coils. A two-dimensional spoiled gradient echo sequence was used for free-breathing imaging, providing coronal plane coverage of the entire lung through 6 to 9 slices [24]. Each slice included 300 dynamic images acquired over a 60-s period. The imaging parameters were as follows: echo time (TE), 0.82 ms; repetition time (TR), 3.0 ms; flip angle (FA), 5°; bandwidth of 1502 Hz per pixel; matrix size, 128 × 128; field of view (FOV) of 500 × 500 mm²; slice thickness, 15 mm. Parallel imaging was applied with an acceleration factor of 2. The total acquisition time for the PREFUL MRI protocol was approximately 6–9 min.

### PREFUL postprocessing and analysis

Datasets were postprocessed with a stand-alone research software (MR Lung v2.2.0, Siemens Healthineers) by two chest radiologists. PREFUL MRI postprocessing process included image registration, segmentation, filtering, and phase sorting as follows [13]:

I. Elastic registration was performed using a mid-ventilation image as a reference. The first 20 images of every PREFUL dataset were discarded to achieve full saturation and ensure a steady state of the stationary tissue.
II. Semiautomatic segmentation of the lung boundaries was performed using a previously trained U-NET convolutional neural network [25]. Manual interaction was applied to make necessary corrections.
III. Images were automatically sorted based on ventilation and perfusion phases. A regional ventilation map and regional flow-volume loops (FVLs) were analyzed as previously described [26–28]. FVLs were derived from voxel-wise time–signal intensity curves acquired during free breathing. Regional ventilation (RVent) was calculated from signal variations over the respiratory cycle, and its first temporal derivative (dRVent/dt) was used to represent airflow. Plotting airflow against RVent generated a voxel-wise FVL, analogous to pulmonary function test flow-volume loops but with spatial resolution. To quantify ventilation dynamics, the mean zero-lag cross-correlation coefficient between voxel-wise FVL signals and a reference FVL obtained from a high-ventilation region (75th–95th percentile of fractional ventilation) was calculated. This FVL correlation metric (FVL-CM) reflects the temporal coherence of regional ventilation throughout the respiratory cycle, with lower values indicating impaired or asynchronous ventilation. The pulmonary perfusion phase was automatically selected from the virtual pulse wave [28]. Normalized perfusion with arbitrary units was calculated (relative to full-blood signal voxels in the central slice) [12].
IV. Mean perfusion was normalized in reference to a full-blood signal region by identifying the lung parenchyma phase via histogram analysis, determining the maximum intensity projection corresponding to large vessels, and dividing the parenchymal phase signal by this normalization value. Mean ventilation was defined as the fractional ventilation of the whole respiratory cycle calculated by the formula $\frac{{S}_{{\mathrm{Mid}}}}{{S}_{{\mathrm{Insp}}}}-\frac{{S}_{{\mathrm{Mid}}}}{{S}_{{\mathrm{Exp}}}}$ with S the signal value at end-inspiration (Insp), end-expiration (Exp), and middle position (Mid). VDPtotal referred to the percentage of total ventilation defect areas, VDPexclusive referred to the percentage of ventilation defects without perfusion defects, QDPtotal referred to the percentage of total perfusion defect areas, QDPexclusive referred to the percentage of perfusion defects without ventilation defects, VQMdefect referred to the percentage of concurrent ventilation and perfusion defects, and VQMnon-defect referred to the percentage of areas with neither ventilation nor perfusion defects. Perfusion defect percentage (QDP) maps, ventilation defect percentage (VDP) maps, and V/Q match maps (VQMs) were automatically calculated. VDP and QDP maps were generated by applying a threshold of 0.9 to the regional FVL correlation coefficient images and 0.2 to the normalized perfusion map [29]. Additionally, QDPchange was defined as the difference between QDP and its cut-off value, with values greater than zero assigned as 1 and others as 0. Similarly, VDPchange was calculated as the difference between VDP and its cut-off value, also categorized as 1 if greater than zero and 0 otherwise. Detailed definitions of these parameters were provided in the Supplementary Table 1.

### Statistical analysis

All statistical analyses were performed using SPSS 20.0 software (IBM) and Origin Pro 2024 SR1 (Origin Lab Corporation). The normality of continuous variables was assessed using the Shapiro-Wilk test. Normally distributed variables were expressed as mean ± standard deviations and compared using an independent samples *t*-test, while non-normally distributed variables were reported as median (interquartile range) and analyzed using the Mann–Whitney *U*-test. Categorical variables were presented as frequencies (percentages) and compared using the chi-square test or Fisher’s exact test. The correlations between variables were evaluated using Spearman’s rank correlation coefficient (ρ), and correlation heatmaps were generated with Origin Pro2024 SR1. Correlation coefficients were defined as high (0.5 < ׀ρ׀ ≤1), moderate (0.3 < ׀ρ׀ ≤0.5), and low (׀ρ׀ ≤ 0.3). In the correlation heatmap, the intensity of the color corresponds to the magnitude of the correlation coefficient (Blue = negative correlation, ρ ≤−0.3; red = positive correlation, ρ ≥ 0.3; white = no or weak correlation. To account for multiple comparisons, *p* values obtained from correlation analyses were adjusted using the Benjamini–Hochberg procedure to control the false discovery rate (FDR). The resulting FDR-adjusted *p* values were used for statistical inference, and an FDR-adjusted *p* value < 0.05 was considered statistically significant. Multivariate linear regression analysis was conducted to examine the relationships between PREFUL MRI-related parameters and PFT, as well as lung lesions on HRCT, after adjusting for demographic factors. A *p* value < 0.05 was considered statistically significant.

## Results

### Clinical characteristics of F-ILD patients

**Fig. 2:**
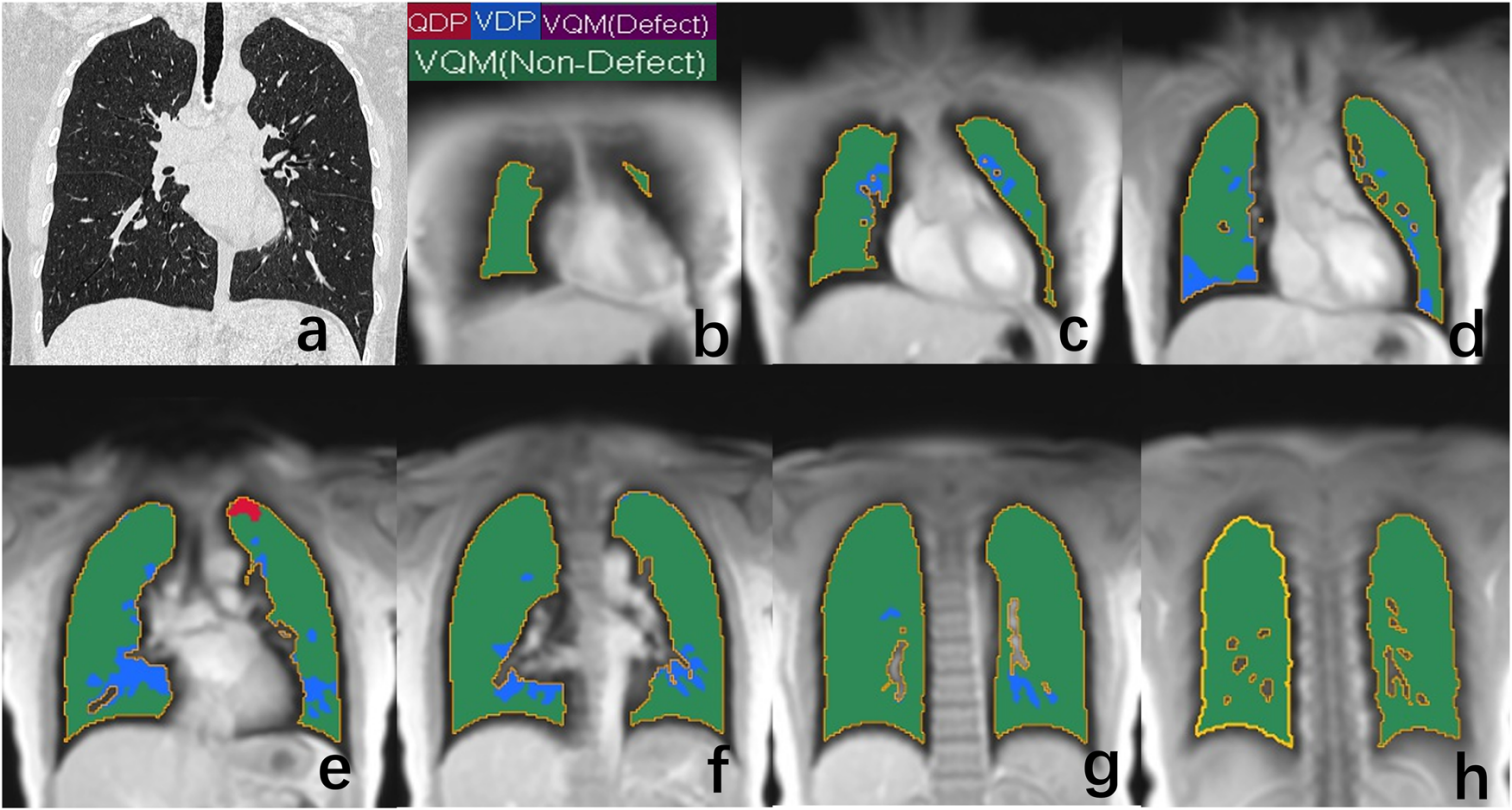
LDCT and PREFUL MRI ventilation/perfusion maps of a 66-year-old healthy male. **a** The coronal LDCT. **b**–**h** The coronal PREFUL MRI Ventilation/Perfusion maps of the whole lung. In ventilation/perfusion maps, red areas indicates QDPexclusive (percentage of areas with perfusion defects but without ventilation defects = 0.3%), blue areas represents VDPexclusive (percentage of areas with ventilation defects but without perfusion defects = 6.6%), green areas corresponds to VQMnon-defect (percentage of areas without perfusion defects or ventilation defects = 93.1%), and purple areas denotes VQMdefect(Percentage of areas with concurrent perfusion and ventilation defects = 0.0%). PREFUL MRI, phase-resolved functional lung magnetic resonance imaging

**Fig. 3:**
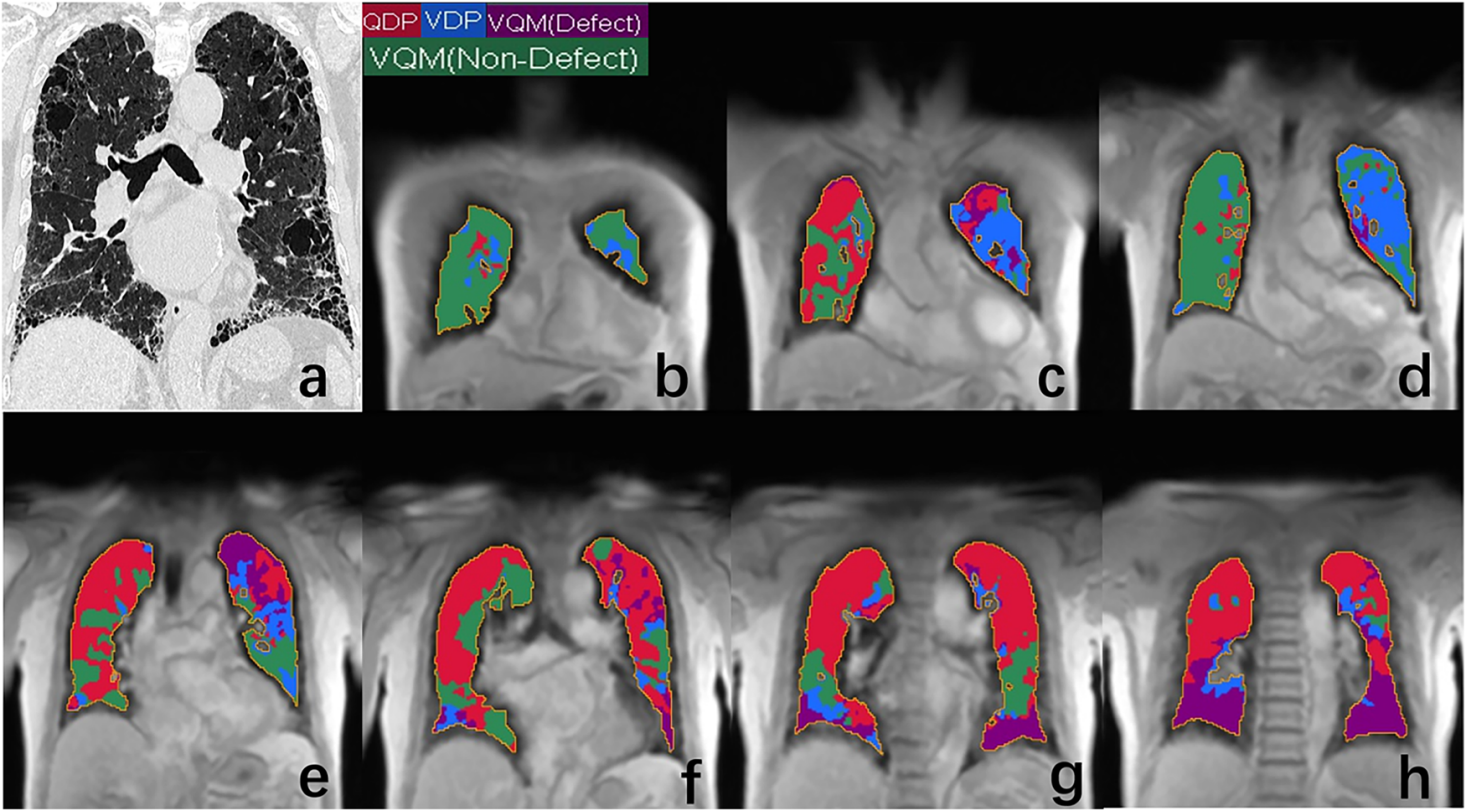
HRCT and PREFUL MRI ventilation/perfusion maps of a 67-year-old male patient with combined pulmonary fibrosis and emphysema. **a** The coronal HRCT indicating both honeycombing and predominantly in the lower zones and emphysema predominantly in the upper zones, **b**–**h** the coronal PREFUL MRI ventilation/perfusion maps of the whole lung. In ventilation/perfusion maps, red areas indicates QDPexclusive (percentage of areas with perfusion defects but without ventilation defects = 39.1%), blue areas represents VDPexclusive (percentage of areas with ventilation defects but without perfusion defects = 15.2%), green areas corresponds to VQMnon-defect (percentage of areas without perfusion defects or ventilation defects = 31.8%), and purple areas denotes VQMdefect (percentage of areas with concurrent perfusion and ventilation defects = 13.8%), prominent in the basal zones of bilateral lung. HRCT, high-resolution CT; PREFUL MRI, phase-resolved functional lung magnetic resonance imaging

A total of 30 F-ILD patients (19 males, mean age: 64.6 ± 9.5 years) were included in the study, with 16 diagnosed with IPF and 14 diagnosed with other types of F-ILD, as confirmed through multidisciplinary discussion. Table 1 shows baseline demographic, pulmonary function, and PREFUL MRI parameters of all participants. No significant differences were observed between the F-ILD group and the healthy control group in terms of sex, age, BMI, systolic blood pressure, and diastolic blood pressure. Figure 2 presents LDCT and PREFUL MRI whole lung perfusion and ventilation maps of a normal volunteer. Figure 3 shows HRCT and PREFUL MRI whole lung perfusion and ventilation maps of a patient with combined pulmonary fibrosis and emphysema, indicating diffused perfusion defects. Compared with normal controls, mean perfusion and VQMnon-defect in F-ILD patients significantly decreased, while mean ventilation, QDPexclusive, and VQMdefect significantly increased. However, the mean FVL Correlation and VDPexclusive between the two groups were comparable.

**Table 1:** Comparison of clinical characteristics between F-ILD patients and healthy controls

Characteristics	Normal controls *N* = 30	F-ILD patients *N* = 30	Χ2/*t*/*U* (*p* value)
Age	63.2 ± 10.5	64.6 ± 9.5	0.068 (0.814)
Sex (male/female)	18/12	19/11	0.071 (0.791)
Smoker (*n*)	0	13	
IPF (*n*)		16	
CPFE (*n*)		1	
CTD-ILD (*n*)		7	
f-NSIP (*n*)		4	
f-HP (*n*)		2	
BMI (Kg/m^2^)	21.3 ± 2.4	22.3 ± 3.1	0.151 (0.269)
SBP (mmHg)	124.1 ± 8.6	122.2 ± 10.4	0.925 (0.139)
DBP (mmHg)	81.4 ± 9.3	79.9 ± 8.6	1.050 (0.677)
Mean perfusion (%)	7.55 (6.43–8.50)	4.60 (3.38–7.10)	−3.579 (< 0.0001)^*^
Mean ventilation (%)	13.95 (10.98–19.38)	18.65 (16.95–22.60)	−3.940 (< 0.0001)^*^
Mean FVL correlation	0.96 (0.95–0.97)	0.97 (0.92–0.98)	−0.127 (0.899)
QDPexclusive (%)	3.65 (2.35–6.63)	15.50 (7.10–25.05)	−4.539 (< 0.0001)^*^
VDPexclusive (%)	7.55 (5.58–10.63)	9.35 (4.60–13.20)	−0.488 (0.626)
VQMnon-defect (%)	87.55 (84.85–90.43)	70.10 (61.60–78.68)	−4.347 (< 0.0001)^*^
VQMdefect (%)	0.15 (0–0.50)	1.35 (0.38–2.70)	−4.109 (< 0.0001)^*^

*IPF* idiopathic pulmonary fibrosis, *CPFE* combined pulmonary fibrosis and emphysema, *CTD-ILD* connective tissue disease associated interstitial lung disease, *f-NSIP* fibrotic nonspecific interstitial pneumonia, *f-HP* fibrotic hypersensitivity pneumonitis, *BMI* body mass index, *SBP* systolic blood pressure, *DBP* diastolic blood pressure, *Mean FVL correlation* mean flow volume loop correlation, *VDPexclusive* percentage of areas with ventilation defects but without perfusion defects, *QDPexclusive* percentage of areas with perfusion defects but without ventilation defects, *VQMdefect* percentage of areas with concurrent perfusion and ventilation defects, *VQMnon-defect* percentage of areas without perfusion defects or ventilation defects

^*^ Statistically significant, *p* < 0.05

### Comparison between IPF and other F-ILD patients

As detailed in Table 2, IPF patients exhibited higher BMI compared to those with other types of F-ILD patients (*p* = 0.023). The CT-based whole-lung percentages of honeycombing, reticulation, consolidation, and fibrotic lesions were comparable between IPF and other F-ILD patients; however, IPF patients displayed less GGO and non-fibrotic lesions (*p* < 0.05). Moreover, pulmonary function parameters, including percentage of predicted forced vital capacity (FVC %pred), percentage of predicted forced expiratory volume in one second (FEV1%pred), FEV1/FVC %pred, TLC-SB %pred, DLCO SB %pred, and DLCO/VA %pred, were similar between the two groups. Furthermore, no significant differences were observed in PREFUL MRI-derived parameters, including QDPexclusive, VDPexclusive, VQMnon-defect, VQMdefect, mean perfusion, mean ventilation, and mean FVL Correlation (all *p* > 0.05) between IPF patients and other F-ILD patients.

**Table 2:** Comparison of clinical data between IPF and other F-ILD patients

Characteristics	IPF patients	Other F-ILD patients	*t*/Χ2/*U* value	*p* value
*n*	16	14		
Sex (male/female)	11/5	8/6	0.433	0707
Age (years)	64.75 ± 9.50	64.36 ± 9.77	0.111	0.912
Smoker (*n*)	8	5	0.621	0.484
BMI (kg/m²)	26.71 ± 2.35	24.55 ± 2.53	2.415	0.023^*^
SBP (mmHg)	122.00 ± 10.71	125.29 ± 18.19	−0.612	0.545
DBP (mmHg)	77.50 ± 8.87	83.07 ± 11.59	−1.489	0.148
Treatment				
Pirfenidone (*n*)	9	1	8.103	0.007^*^
Nintedanib (*n*)	4	2	0.536	0.657
Other treatment (*n*)	5	10	4.821	0.066
HRCT quantitative parameters				
The whole-lung percentage of honeycombing (%)	1.00 (0–5.50)	0.50 (0–1.50)	−1.325	0.208
The whole-lung percentage of emphysema (%)	0 (0–1.75)	0 (0–0.25)	−1.317	0.275
The whole-lung percentage of reticulation (%)	12.00 (5.25–16.00)	12.00 (4.00–23.50)	−0.083	0.951
The whole-lung percentage of GGO (%)	0 (0–0)	1.00 (0–2.75)	−2.378	0.043^*^
The whole-lung percentage of consolidation (%)	0	0 (0–1.00)	−2.568	0.101
The whole-lung percentage of fibrotic lesions (%)	14.50 (5.25–21.75)	11.50 (4.00–23.75)	−0.250	0.822
The whole-lung percentage of non-fibrotic lesion (%)	0 (0–0)	1.00 (0–2.75)	−2.378	0.043^*^
Total interstitial lesion burden (%)	16.50 (5.50–26.25)	18.00 (8.75–27.75)	−0.354	0.728
PFTs				
VC %pred	88.10 (72.60–95.68)	77.75 (59.38–102.85)	−1.039	0.299
FVC %pred	89.45 (74.98–97.43)	78.55 (60.65–106.15)	−1.039	0.299
FEV1%pred	74.25 (64.03–99.73)	92.60 (80.13–101.35)	−1.455	0.146
FEV1/FVC %pred	98.45 (93.03–102.18)	95.35 (88.65–104.83)	−0.270	0.787
TLC-SB %pred	74.60 (61.05–82.60)	73.05 (57.68–92.58)	−0.104	0.917
DLCO SB %pred	63.95 (44.50–71.60)	53.15 (39.83–73.68)	−0.707	0.480
DLCO/VA %pred	87.40 (68.28–97.53)	87.15 (67.90–96.85)	0	1.000
PREFUL MRI				
Mean perfusion (%)	4.50 (3.45–7.55)	4.65 (3.05–6.93)	−0.645	0.525
Mean ventilation (%)	18.45 (16.80–21.13)	22.00 (17.38–26.13)	−1.331	0.193
QDPexclusive (%)	15.5 (4.55–27.15)	15.9 (8.10–28.23)	−0.478	0.633
VDPexclusive (%)	7.45 (3.40–12.23)	10.95 (6.03–14.70)	−1.123	0.262
VQMdefect (%)	1.70 (0.35–3.25)	1.10 (0.38–2.55)	−0.083	0.934
VQMnon-defect (%)	72.40 (60.80–88.53)	69.80 (58.48–78.13)	−0.707	0.480
Mean FVL correlation	0.97 (0.95–0.98)	0.96 (0.92–0.98)	−0.461	0.667

*BMI* body mass index, *SBP* systolic blood pressure, *DBP* diastolic blood pressure, *GGO* ground-glass opacity, *FVC %pred* percentage of predicted forced vital capacity, *FEV1%pred* percentage of predicted forced expiratory volume in one second, *FEV1/FVC %pred* percentage of predicted forced expiratory volume in one second divided by forced vital capacity, *VC %pred* percentage of predicted vital capacity, *TLC-SB %pred* percentage of predicted single breath total lung capacity, *DLCO SB %pred* percentage of predicted single breath diffusing capacity for carbon monoxide, *DLCO/VA %pred* percentage of predicted diffusing capacity for carbon monoxide divided by the alveolar volume, *QDPexclusive* percentage of areas with perfusion defects but without ventilation defects, *VDPexclusive* percentage of areas with ventilation defects but without perfusion defects, *VQMdefect* percentage of areas with concurrent perfusion and ventilation defects, *VQMnon-defect* percentage of areas without perfusion defects or ventilation defects, *mean FVL correlation* mean flow volume loop correlation

^*^ Statistically significant, *p* < 0.05

### Correlation of PREFUL MRI-related parameters and PFTs in F-ILD Patients

**Fig. 4:**
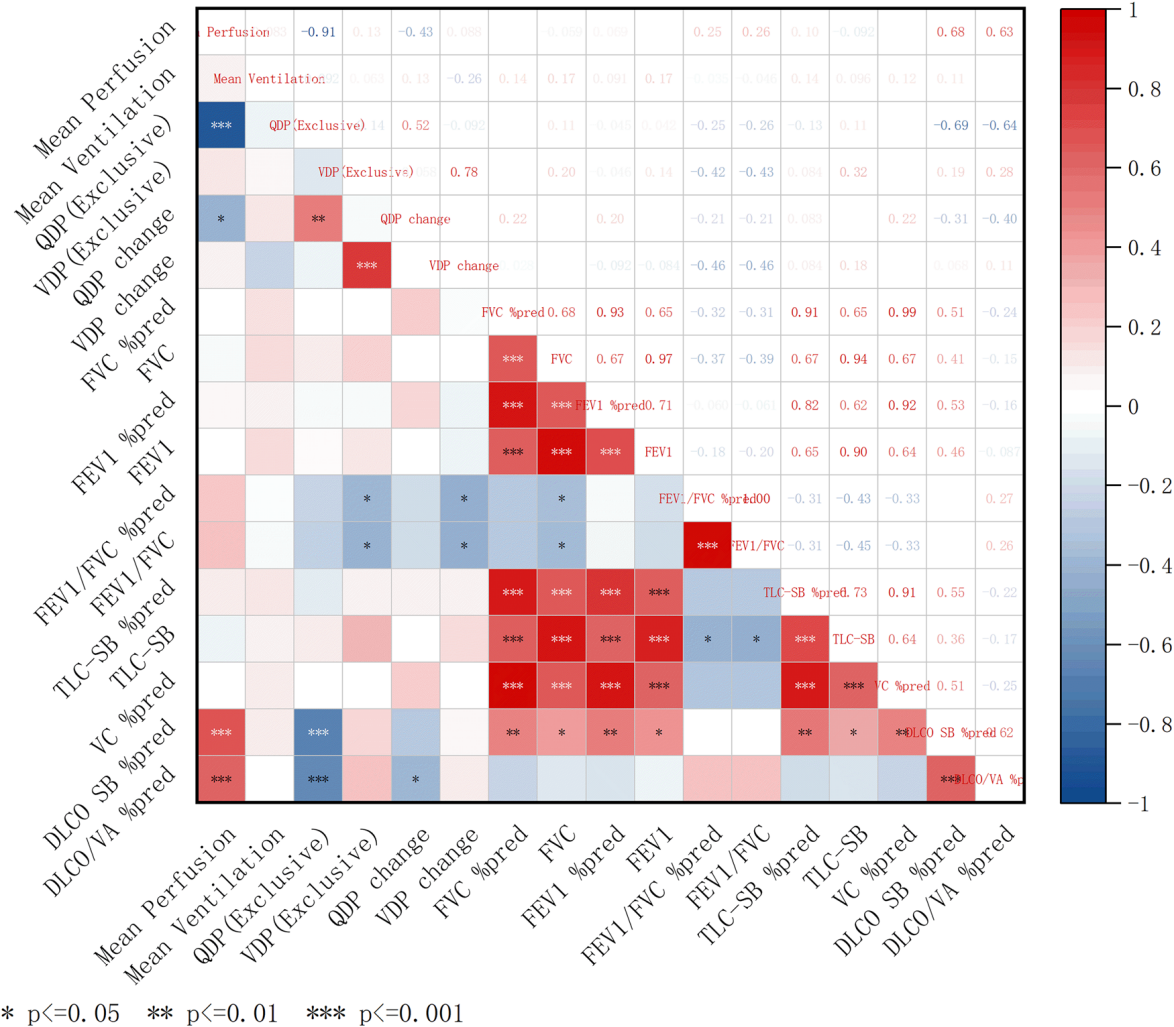
Correlation heatmap analysis between PREFUL MRI-related parameters and pulmonary function in F-ILD patients. Correlations were calculated with Spearman’s rank test. In a correlation heatmap, the intensity of the color corresponds to the magnitude of the correlation coefficient (blue = negative correlation, ρ ≤ −0.3; red = positive correlation, ρ ≥ 0.3; white = no or weak correlation. VDPexclusive, percentage of areas with ventilation defects but without perfusion defects; QDPexclusive, percentage of areas with perfusion defects but without ventilation defects; QDPchange was defined as the difference between QDP and its cut-off value, with values greater than zero assigned as 1 and others as 0. Similarly, VDPchange was calculated as the difference between VDP and its cut-off value, also categorized as 1 if greater than zero and 0 otherwise; FVC %pred, percentage of predicted forced vital capacity; FEV1%pred, percentage of predicted forced expiratory volume in one second; VC %pred, percentage of predicted vital capacity; TLC %pred, percentage of predicted total lung capacity; DLCO SB %pred, percentage of predicted single breath diffusing capacity for carbon monoxide; DLCO/VA %pred, percentage of predicted diffusing capacity for carbon monoxide divided by the alveolar volume

The correlation Heatmap (Fig. 4) illustrates significant correlations between PREFUL MRI and PFTs parameters in patients with F-ILD. Mean perfusion was significantly positively correlated with both DLCO SB %pred (ρ = 0.682, *p* < 0.001) and DLCO/VA %pred (ρ = 0.634, *p* < 0.001). Conversely, QDPexclusive showed a significant inverse correlation with DLCO SB %pred (ρ = −0.688, *p* < 0.001) and DLCO/VA %pred (ρ = −0.645, *p* < 0.001). Notably, QDPchange demonstrates a moderate negative relationship with DLCO/VA %pred (ρ = −0.404, *p* = 0.027). Both VDPexclusive (ρ = −0.417, *p* = 0.022) and VDPchange (ρ = −0.460, *p* = 0.011) exhibited significant negative correlations with FEV1/FVC %pred. After adjustment for multiple correlations using the Benjamini-Hochberg method (false discovery rate, FDR α = 0.05), mean perfusion remained significantly positively correlated with both DLCO %pred (ρ = 0.682, FDR-adjusted *p* = 0.001) and DLCO/VA %pred (ρ = 0.634, FDR-adjusted *p* = 0.003). QDPchange remained significantly negatively correlated with DLCO %pred (ρ = −0.688, FDR-adjusted *p* = 0.002) and DLCO/VA %pred (ρ = −0.645, FDR-adjusted *p* = 0.003). (Supplementary Table 2).

### Correlation of PREFUL MRI-related parameters and lung lesions on HRCT

**Fig. 5:**
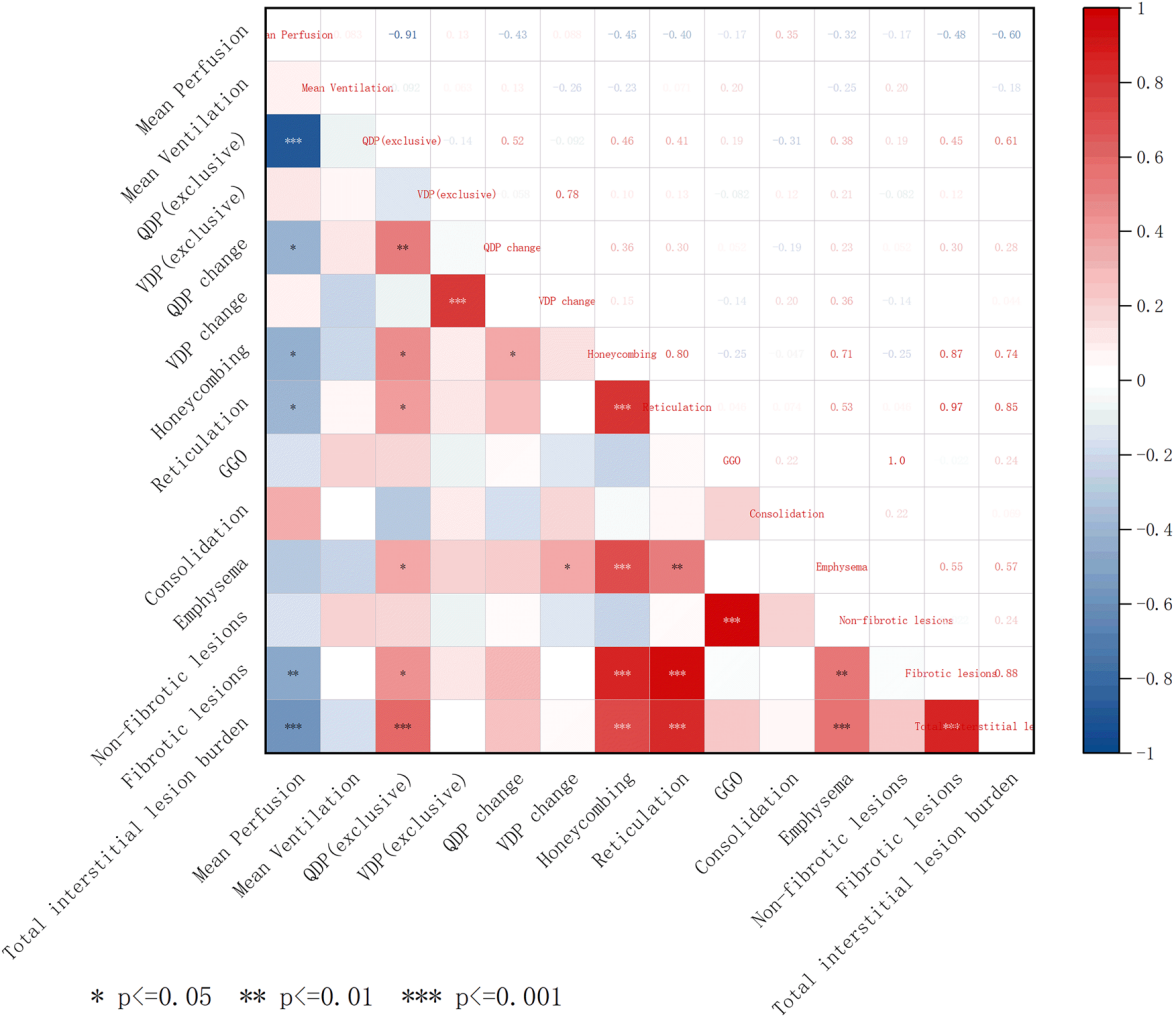
Correlation heatmap analysis between PREFUL MRI-related parameters and lung lesions on HRCT in F-ILD patients. Correlations were calculated with Spearman’s rank test. In a correlation heatmap, the intensity of the color corresponds to the magnitude of the correlation coefficient (Blue = negative correlation, ρ ≤ −0.3; red = positive correlation, ρ ≥ 0.3; white = no or weak correlation. VDPexclusive, percentage of areas with ventilation defects but without perfusion defects; QDPexclusive, percentage of areas with perfusion defects but without ventilation defects; QDPchange, the difference between QDP and its cut-off value, with values greater than zero assigned as 1 and others as 0. Similarly, VDPchange was calculated as the difference between VDP and its cut-off value, also categorized as 1 if greater than zero and 0 otherwise; GGO, ground-glass opacity

Figure 5 is the correlation heatmap of PREFUL MRI-related parameters and Lung Lesions on HRCT, mean perfusion negatively correlated with the whole-lung percentage of honeycombing (ρ = −0.447, *p* = 0.013), reticulation (ρ = −0.404, *p* = 0.027), fibrotic lesions (ρ = −0.481, *p* = 0.007), and total interstitial lesion burden (ρ = −0.600, *p* < 0.001). In contrast, QDPexclusive exhibited positive correlations with the whole-lung percentage of honeycombing (ρ = 0.461, *p* = 0.010), reticulation (ρ = 0.410, *p* = 0.024), and fibrotic lesions (ρ = 0.446, *p* = 0.013), as well as total interstitial lesion burden (ρ = 0.610, *p* < 0.001) and emphysema (ρ = 0.379, *p* = 0.039). Additionally, QDPchange positively correlated with the whole-lung percentage of honeycombing (ρ = 0.362, *p* = 0.049), while VDPchange showed a comparable association with the whole-lung percentage of emphysema (ρ = 0.363, *p* = 0.049). After adjustment for multiple correlations using the Benjamini-Hochberg method (FDR α = 0.05), mean perfusion remained significantly negatively correlated with total interstitial lesion burden (ρ = −0.600, FDR-adjusted *p* < 0.001). Conversely, QDPexclusive remained significantly positively correlated with total interstitial lesion burden (ρ = 0.610, FDR-adjusted *p* < 0.001) (see Supplementary Table 3 for additional details).

### Correlation of lung lesions on HRCT and PFTs

**Fig. 6:**
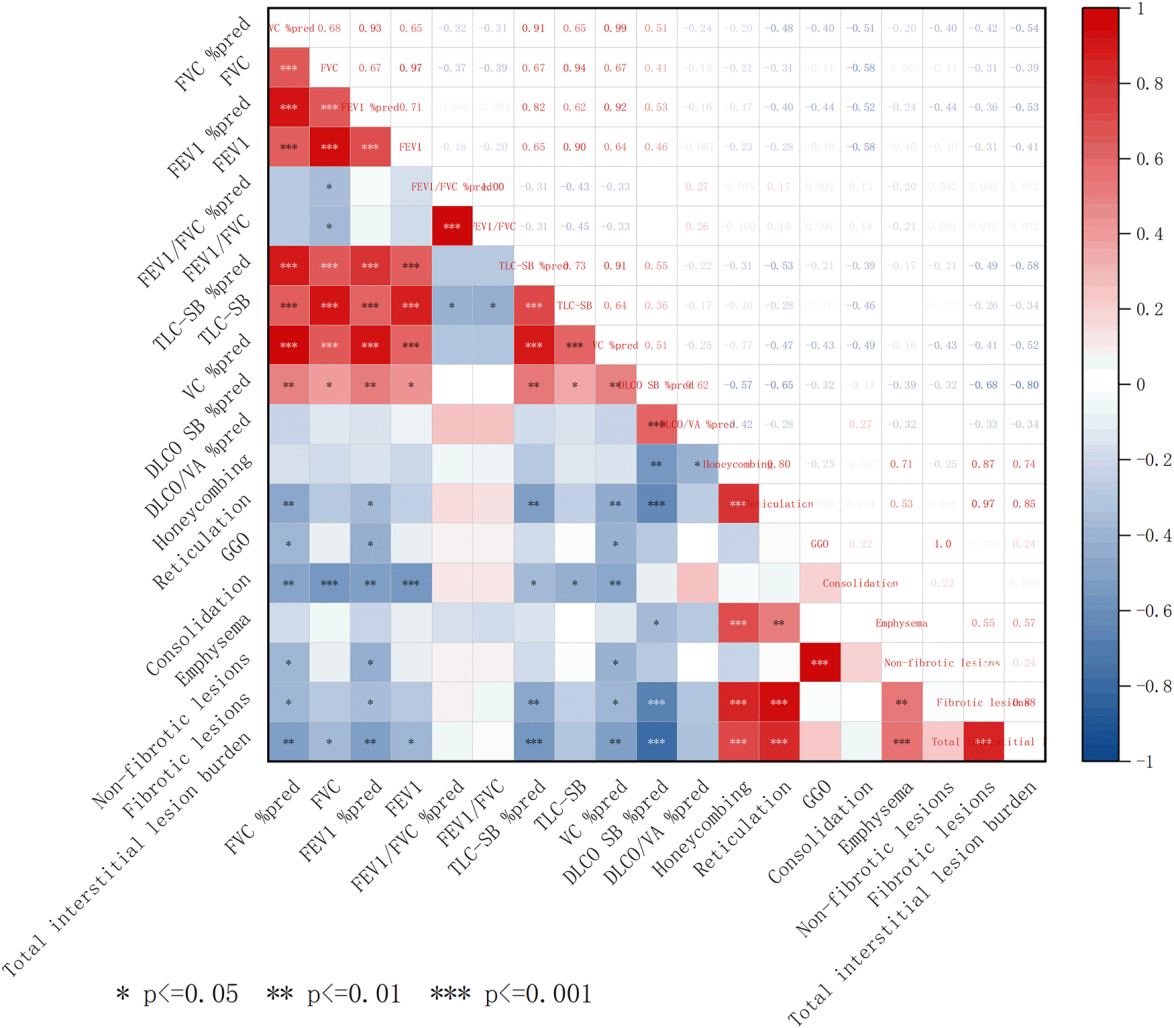
Correlation heatmap analysis between lung lesions on HRCT and PFTs in F-ILD patients. Notes: Correlations were calculated with Spearman’s rank test. In a correlation heatmap, the intensity of the color corresponds to the magnitude of the correlation coefficient (blue = negative correlation, ρ ≤ −0.3; red = positive correlation, ρ ≥ 0.3; white = no or weak correlation. FVC %pred, percentage of predicted forced vital capacity; FEV1%pred, percentage of predicted forced expiratory volume in one second; VC %pred, percentage of predicted vital capacity; TLC %pred, percentage of predicted total lung capacity; DLCO SB %pred, percentage of predicted single breath diffusing capacity for carbon monoxide; DLCO/VA %pred, percentage of predicted diffusing capacity for carbon monoxide divided by the alveolar volume; GGO, ground-glass opacity

As shown in Fig. 6, the percentage of total interstitial lesion burden on HRCT has a significantly negative correlation with FVC %pred (ρ = −0.540, *p* = 0.002), TLC SB %pred (ρ = −0.338, *p* = 0.001), DLCO SB %pred (ρ = −0.801, *p* < 0.001), and VC %pred (ρ = −0.522, *p* = 0.003), as well as FEV1%pred (ρ = −0.531, *p* = 0.003). Disaggregating by imaging findings, the reticular opacity percentage negatively correlated with FVC %pred (ρ = −0.480, *p* = 0.007), TLC SB %pred (ρ = −0.527, *p* = 0.003), DLCO SB %pred (ρ = −0.651, *p* < 0.001), and VC %pred (ρ = −0.470, *p* = 0.009), and with FEV1%pred (ρ = −0.399, *p* = 0.029). The honeycombing percentage is significantly negatively correlated with DLCO SB %pred (ρ = −0.566, *p* = 0.001) and negatively correlated with DLCO/VA %pred (ρ = −0.422, *p* = 0.020). The consolidation percentage is significantly negatively correlated with FVC %pred (ρ = −0.515, *p* = 0.004), FEV1%pred (ρ = −0.525, *p* = 0.003), and VC %pred (ρ = −0.495, *p* = 0.005), and negatively correlated with TLC SB %pred (ρ = −0.393, *p* = 0.032). Subsequently, the GGO percentage is negatively correlated with FVC %pred (ρ = −0.405, *p* = 0.026), FEV1%pred (ρ = −0.442, *p* = 0.015), and VC %pred (ρ = −0.430, *p* = 0.018).

After adjustment for multiple correlations using the Benjamini-Hochberg method (FDR α = 0.05), the whole-lung percentage of honeycombing, reticulation, fibrotic lesions, and the percentage of total interstitial lesion burden remained significantly negatively correlated with DLCO SB %pred (ρ = −0.566, FDR-adjusted *p* = 0.014; ρ = −0.651, FDR-adjusted *p* < 0.001; ρ = −0.684, FDR-adjusted *p* < 0.001; ρ = −0.801, FDR-adjusted *p* < 0.001). The whole-lung percentage of consolidation remained significantly negatively correlated with FEV1 (ρ = −0.578, FDR-adjusted *p* = 0.014), FEV1%pred (ρ = −0.525, FDR-adjusted *p* = 0.028), FVC (ρ = −0.576, FDR-adjusted *p* = 0.014), FVC %pred (ρ = −0.515, FDR-adjusted *p* = 0.033), and VC %pred (ρ = −0.495, FDR-adjusted *p* = 0.039) (see Supplementary Table 4 for additional details).

### Multivariate analysis of PREFUL MRI-related parameters and PFTs, lung lesions

After adjustment for demographic factors including age, sex, and BMI (Table 3), each 1% increase in mean perfusion was independently associated with a 0.578-percentage-point rise in DLCO SB %pred (95% CI: 1.33–5.36; *p* = 0.002) and a 0.660-standard-unit increase in DLCO/VA (95% CI: 1.97–5.90; *p* < 0.001). Conversely, every 1% increment in QDPexclusive exclusively reduced DLCO SB %pred by 0.576 percentage points and DLCO/VA %pred by 0.706 standard units. Moreover, a 1% rise in QDPexclusive added approximately 0.572 percentage points to the whole-lung percentage of non-fibrotic lesions and 0.553 percentage points to total interstitial lesion burden. These findings demonstrate that, even after controlling for age, sex, and BMI, PREFUL-MRI perfusion metrics (mean perfusion and QDPexclusive) remain significant, independent predictors of gas-exchange capacity and fibrotic burden, indicating that they provide additional, irreplaceable pathological information beyond simple demographics.

**Table 3:** Multivariate linear regression analysis of PREFUL MRI-related parameters with PFT and lung lesions on HRCT after adjusting for demographics

Dependent variable	Independent variable	β	95% CI	*p* value	Adjusted *R*^2^
DLCO SB %pred	Mean perfusion (%)	0.578	(1.330, 5.358)	0.002^*^	0.302
	Mean ventilation (%)	−0.099	(−1.767, 1.046)	0.603	−0.001
	QDPexclusive (%)	−0.576	(−1.275, −0.251)	0.005^*^	0.257
	QDPchange	−0.297	(−46.349, 5.848)	0.123	0.079
	VDPexclusive (%)	0.434	(0.176, 2.084)	0.022^*^	0.177
	VDPchange	0.306	(−3.085, 29.121)	0.109	0.086
DLCO/VA %pred	Mean perfusion (%)	0.660	(1.968, 5.895)	< 0.001^*^	0.373
	Mean ventilation (%)	−0.028	(−1.574, 1.369)	0.887	−0.035
	QDPexclusive (%)	−0.706	(−1.448, −0.476)	< 0.001^*^	0.367
	QDPchange	−0.420	(−55.383, −3.656)	0.027^*^	0.145
	VDPexclusive (%)	0.605	(0.733, 2.505)	0.001^*^	0.328
	VDPchange	0.253	(−6.010, 28.112)	0.195	0.030
FEV1	Mean perfusion (%)	−0.008	(−0.075, 0.071)	0.963	0.271
	Mean ventilation (%)	0.055	(−0.035, 0.050)	0.733	0.275
	QDPexclusive (%)	−0.048	(−0.020, 0.016)	0.799	0.273
	QDPchange	0.146	(−0.457, 1.164)	0.378	0.293
	VDPexclusive (%)	−0.123	(−0.043, 0.020)	0.467	0.286
	VDPchange	−0.059	(−0.597, 0.419)	0.722	0.275
FEV1/FVC	Mean perfusion (%)	0.069	(−0.661, 0.946)	0.719	0.123
	Mean ventilation (%)	−0.031	(−0.510, 0.429)	0.862	0.120
	QDPexclusive (%)	−0.255	(−0.312, 0.072)	0.211	0.171
	QDPchange	−0.222	(−14.215, 3.433)	0.220	0.169
	VDPexclusive (%)	−0.135	(−0.473, 0.222)	0.465	0.137
	VDPchange	−0.301	(−9.876, 0.766)	0.090	0.213
FEV1/FVC %pred	Mean perfusion (%)	0.067	(−0.791, 1.117)	0.728	0.105
	Mean ventilation (%)	−0.025	(−0.596, 0.519)	0.889	0.102
	QDPexclusive (%)	−0.263	(−0.374, 0.082)	0.200	0.157
	QDPchange	−0.228	(−16.975, 3.951)	0.212	0.154
	VDPexclusive (%)	−0.138	(−0.563, 0.262)	0.459	0.120
	VDPchange	−0.307	(−11.768, 0.849)	0.087	0.198
FEV1%pred	Mean perfusion (%)	0.044	(−2.527, 3.086)	0.839	−0.106
	Mean ventilation (%)	0.006	(−1.616, 1.662)	0.977	−0.108
	QDPexclusive (%)	−0.137	(−0.888, 0.487)	0.554	−0.093
	QDPchange	0.203	(−15.720, 46.447)	0.319	−0.066
	VDPexclusive (%)	−0.109	(−1.533, 0.904)	0.601	−0.096
	VDPchange	−0.044	(−21.677, 17.548)	0.830	−0.106
FVC	Mean perfusion (%)	−0.041	(−0.112, 0.089)	0.820	0.239
	Mean ventilation (%)	0.050	(−0.050, 0.067)	0.763	0.240
	QDPexclusive (%)	0.067	(−0.020, 0.029)	0.727	0.241
	QDPchange	0.166	(−0.573, 1.652)	0.328	0.266
	VDPexclusive (%)	−0.114	(−0.058, 0.029)	0.510	0.250
	VDPchange	−0.004	(−0.711, 0.693)	0.979	0.238
FVC %pred	Mean perfusion (%)	−0.002	(−3.139, 3.112)	0.993	−0.102
	Mean ventilation (%)	0.020	(−1.732, 1.914)	0.919	−0.102
	QDPexclusive (%)	−0.009	(−0.784, 0.756)	0.970	−0.102
	QDPchange	0.239	(−14.163, 54.471)	0.238	−0.044
	VDPexclusive (%)	−0.064	(−1.565, 1.156)	0.760	−0.098
	VDPchange	0.056	(−18.865, 24.754)	0.784	−0.099
TLC-SB	Mean perfusion (%)	0.057	(−0.115, 0.156)	0.756	0.200
	Mean ventilation (%)	0.014	(−0.076, 0.082)	0.934	0.197
	QDPexclusive (%)	0.031	(−0.031, 0.036)	0.875	0.198
	QDPchange	0.066	(−1.246, 1.811)	0.707	0.202
	VDPexclusive (%)	0.008	(−0.058, 0.061)	0.963	0.197
	VDPchange	0.108	(−0.653, 1.231)	0.534	0.209
TLC-SB %pred	Mean perfusion (%)	0.094	(−1.791, 2.787)	0.658	−0.088
	Mean ventilation (%)	−0.054	(−1.518, 1.159)	0.785	−0.093
	QDPexclusive (%)	−0.015	(−0.585, 0.547)	0.946	−0.096
	QDPchange	0.115	(−18.595, 32.935)	0.572	−0.083
	VDPexclusive (%)	0.046	(−0.892, 1.111)	0.824	−0.094
	VDPchange	0.169	(−9.291, 22.388)	0.403	−0.067
VC %pred	Mean perfusion (%)	0.009	(−2.952, 3.081)	0.965	−0.103
	Mean ventilation (%)	0.009	(−1.720, 1.799)	0.964	−0.103
	QDPexclusive (%)	−0.017	(−0.770, 0.716)	0.941	−0.103
	QDPchange	0.237	(−13.823, 52.450)	0.242	−0.045
	VDPexclusive (%)	−0.059	(−1.498, 1.129)	0.775	−0.099
	VDPchange	0.069	(−17.542, 24.527)	0.736	−0.098
The whole-lung percentage of GGO (%)	Mean perfusion (%)	−0.125	(−1.534, 0.836)	0.550	−0.044
	Mean ventilation (%)	−0.132	(−0.920, 0.460)	0.500	−0.040
	QDPexclusive (%)	0.572	(0.110, 0.620)	0.007*	0.206
	QDPchange	0.572	(0.110, 0.620)	0.007*	0.206
	VDPexclusive (%)	−0.095	(−0.637, 0.400)	0.642	−0.050
	VDPchange	−0.137	(−11.076, 5.451)	0.490	−0.039
The whole-lung percentage of emphysema (%)	Mean perfusion (%)	−0.282	(−0.831, 0.141)	0.157	0.085
	Mean ventilation (%)	−0.176	(−0.425, 0.155)	0.348	0.044
	QDPexclusive (%)	0.317	(−0.031, 0.208)	0.139	0.092
	QDPchange	0.116	(−3.995, 7.332)	0.550	0.024
	VDPexclusive (%)	0.004	(−0.218, 0.222)	0.985	0.010
	VDPchange	0.337	(−0.294, 6.337)	0.072	0.128
The whole-lung percentage of honeycombing (%)	Mean perfusion (%)	−0.233	(−1.011, 0.254)	0.229	0.123
	Mean ventilation (%)	−0.106	(−0.485, 0.269)	0.560	0.085
	QDPexclusive (%)	0.254	(−0.061, 0.250)	0.225	0.124
	QDPchange	0.084	(−5.701, 8.915)	0.655	0.080
	VDPexclusive (%)	0.003	(−0.282, 0.286)	0.988	0.072
	VDPchange	0.133	(−2.916, 6.082)	0.476	0.091
The whole-lung percentage of consolidation (%)	Mean perfusion (%)	0.033	(−0.099, 0.116)	0.870	0.052
	Mean ventilation (%)	−0.073	(−0.074, 0.050)	0.695	0.057
	QDPexclusive (%)	−0.107	(−0.033, 0.020)	0.616	0.060
	QDPchange	−0.452	(−2.479, −0.342)	0.012^*^	0.260
	VDPexclusive (%)	0.046	(−0.041, 0.052)	0.812	0.053
	VDPchange	0.085	(−0.581, 0.913)	0.652	0.059
The whole-lung percentage of reticulation (%)	Mean perfusion (%)	−0.280	(−1.495, 0.260)	0.160	0.086
	Mean ventilation (%)	0.205	(−0.236, 0.804)	0.271	0.058
	QDPexclusive (%)	0.096	(−0.176, 0.272)	0.661	0.020
	QDPchange	0.223	(−4.226, 15.824)	0.245	0.630
	VDPexclusive (%)	−0.047	(−0.444, 0.351)	0.811	0.015
	VDPchange	−0.085	(−7.720, 4.983)	0.661	0.020
The whole-lung percentage of non-fibrotic lesions (%)	Mean perfusion (%)	−0.125	(−1.534, 0.836)	0.550	−0.044
	Mean ventilation (%)	−0.132	(−0.920, 0.460)	0.500	−0.040
	QDPexclusive (%)	0.572	(0.110, 0.620)	0.007^*^	0.206
	QDPchange	0.042	(−12.075, 14.838)	0.835	−0.057
	VDPexclusive (%)	−0.095	(−0.637, 0.400)	0.642	−0.050
	VDPchange	−0.137	(−11.076, 5.451)	0.490	−0.039
The whole-lung percentage of fibrotic lesions (%)	Mean perfusion (%)	−0.302	(−2.300, 0.270)	0.116	0.160
	Mean ventilation (%)	0.077	(−0.622, 0.946)	0.675	0.081
	QDPexclusive (%)	0.200	(−0.173, 0.480)	0.342	0.107
	QDPchange	0.182	(−7.704, 22.166)	0.329	0.108
	VDPexclusive (%)	−0.024	(−0.625, 0.551)	0.899	0.075
	VDPchange	0.014	(−9.081, 9.767)	0.941	0.075
Total interstitial lesion burden (%)	Mean perfusion (%)	−0.360	(−3.458, 0.097)	0.063	0.162
	Mean ventilation (%)	−0.069	(−1.307, 0.906)	0.712	0.045
	QDPexclusive (%)	0.553	(0.186, 0.994)	0.006^*^	0.287
	QDPchange	0.164	(−12.106, 30.197)	0.387	0.068
	VDPexclusive (%)	−0.074	(−0.983, 0.671)	0.702	0.046
	VDPchange	0.017	(−12.702, 13.881)	0.928	0.040

*PFT* pulmonary function test, *DLCO SB %pred* percentage of predicted single breath diffusing capacity for carbon monoxide, *DLCO/VA %pred* percentage of predicted diffusing capacity for carbon monoxide divided by the alveolar volume, *FEV1/FVC %pred* percentage of predicted forced expiratory volume in one second divided by forced vital capacity, *QDPexclusive* percentage of areas with perfusion defects but without ventilation defects, *VDPexclusive* percentage of areas with ventilation defects but without perfusion defects, *QDPchange* the difference between QDP and its cut-off value, with values greater than zero assigned as 1 and others as 0. Similarly, VDPchange was calculated as the difference between VDP and its cut-off value, also categorized as 1 if greater than zero and 0 otherwise

^*^ Statistically significant, *p* < 0.05

## Discussion

This study firstly utilized PREFUL MRI to assess pulmonary perfusion and ventilation in F-ILD patients, and there are several important findings: (I). F-ILD patients exhibited significantly reduced mean perfusion compared to normal controls, indicating impaired pulmonary blood flow. (II) There was a notable increase in both QDPexclusive and VQMdefect in F-ILD patients, highlighting significant ventilation and perfusion defects. But Mean FVL Correlation and VDPexclusive were comparable between F-ILD patients and normal controls, indicating that these parameters may not be as sensitive to the disease state of F-ILD. (III) PREFUL MRI parameters were comparable between IPF and other types of F-ILD, suggesting similar underlying pathological processes in terms of perfusion and ventilation. However, the percentage of GGO on HRCT was more prevalent in other types of F-ILD patients than in IPF, indicating potential differences in disease presentation or progression. (IV) Mean perfusion and QDPexclusive in F-ILD patients were significantly correlated with key pulmonary function indices such as DLCO, SB, and FEV1/FVC, indicating that PREFUL MRI parameters can provide valuable insights into the functional impact of the disease, potentially serving as surrogate markers for pulmonary function. (V) Perfusion-related parameters on PREFUL MRI were significantly correlated with the whole-lung percentage of fibrotic lesions on HRCT, indicating that MRI can capture the extent of structural lung damage, underscoring the potential of PREFUL MRI as a non-invasive imaging modality to assess disease severity and monitor progression of F-ILD.

F-ILD patients show marked perfusion impairment, lower mean perfusion, and higher QDPexclusive indicating vascular damage and small vessel loss that may drive IPF progression [22, 30, 31]. Counterintuitively, F-ILD patients demonstrated significantly elevated Mean Ventilation versus controls. Pulmonary fibrosis may cause tissue destruction and remodeling that leads to uneven ventilation, with PREFUL MRI detecting elevated Mean ventilation in only healthy regions. Compensatory increases in ventilation and higher respiratory rates due to dyspnea may further boost mean values without improving overall gas exchange. Meanwhile, reduced VQMnon-defect and increased VQMdefect underscore a significant ventilation-perfusion mismatch contributing to oxygenation dysfunction.

In patients with COPD or asthma, mean FVL Correlation significantly decreases, and VDPexclusive shows a significant increase, indicating that these metrics are sensitive airway biomarkers [9, 11, 15]. Specifically, a high FVL correlation reflected healthy ventilation dynamics, whereas a low correlation reflected abnormal ventilation dynamics [16]. In contrast, Mean FVL Correlation and VDPexclusive values in F-ILD patients were similar to those of healthy controls. When comparing IPF to other types of F-ILD, the IPF group exhibited lower GGO on HRCT, which is consistent with the typical pathological features of IPF, characterized by predominant fibrosis with relatively less inflammation. However, no significant differences were observed in PFTs or PREFUL MRI parameters between the two groups, indicating that while fibrosis influences lung function, its etiology may have a limited impact on these functional parameters.

Previous studies have demonstrated a robust correlation between PREFUL MRI-derived ventilation parameters and pulmonary function outcomes in patients with COPD [32], as well as a significant association between QDP and FEV1%pred in patients with cystic fibrosis [33]. Recently, a Meta-analysis indicated that PREFUL MRI was a promising imaging modality for the assessment of lung function, when compared with dynamic contrast- enhanced MRI and 129Xe MRI [34]. Our study revealed that key PREFUL MRI parameters, such as mean perfusion, QDPexclusive, and VDPexclusive, were significantly correlated with PFTs in patients with F-ILD. These findings indicate that PREFUL MRI parameters can accurately reflect lung diffusion function, providing a non-invasive method for assessing gas exchange in F-ILD patients. DLCO is a key indicator of alveolar-capillary gas exchange capacity and reflects pulmonary microvascular function. The significant positive correlation between mean perfusion and DLCO SB %pred suggests that higher pulmonary perfusion is associated with improved gas exchange efficiency. Since DLCO/VA accounts for alveolar volume, its strong correlation with mean perfusion further reinforces the relationship between pulmonary perfusion and gas exchange capacity, indicating that higher perfusion corresponds to better alveolar diffusion function. Additionally, VDPexclusive showed a significant negative correlation with FEV1/FVC %pred, reflecting the association between ventilation impairment and airflow limitation.

In terms of fibrosis features on HRCT, mean perfusion negatively correlated with characteristic fibrotic patterns, including honeycombing, reticulation, fibrotic lesions (combined honeycombing and reticulation), and total interstitial lesion burden. Conversely, QDPexclusive showed significant positive correlations with these CT features. Current CT-based quantitative parameters, while useful, are limited by radiation exposure and may not capture functional changes as sensitively as PREFUL MRI. Our study shows that PREFUL MRI parameters have higher regression coefficients compared with CT-based fibrosis scores, indicating that PREFUL MRI may be more sensitive in detecting early disease progression.

Our multivariate analysis reveals that PREFUL MRI parameters, specifically mean perfusion and QDPexclusive, remain significant predictors of pulmonary function and fibrotic burden, independent of age, sex, and BMI. This is a crucial finding, as it indicates that PREFUL MRI captures pathophysiological signals that are distinct from mere demographic variations. For instance, mean perfusion independently explains nearly 40% of the variance in DLCO/VA, a key marker of gas exchange capacity. This suggests that PREFUL MRI can differentiate between physiological aging and pathological fibrosis progression, offering a more nuanced understanding of disease dynamics.

The clinical relevance of these findings is substantial. The regression coefficients derived from our analysis can be translated into actionable thresholds for clinical decision-making. For example, a 10% increase in QDPexclusive is associated with about 7.06% reduction in DLCO/VA, which is comparable to the average annual decline observed in F-ILD. This threshold can serve as an early warning signal, allowing clinicians to initiate more aggressive treatment strategies or increase monitoring frequency.

Despite these promising results, our study has several limitations that need to be addressed. First, the relatively small sample size in a single-center design may limit the generalizability of the findings. Future multicenter studies with larger cohorts are necessary to further validate the clinical utility of PREFUL MRI in the assessment of ILD. Second, as a cross-sectional study, it lacks longitudinal data, precluding an evaluation of the potential value of PREFUL MRI parameters in predicting disease progression and assessing treatment responses. Future studies should include serial imaging to establish the temporal dynamics of these parameters. Third, the accuracy of PREFUL MRI in fibrosis staging also requires further investigation. Although PREFUL MRI offers significant advantages in functional assessment, there is still room for improvement in image resolution and scan time. Future research should focus on enhancing image quality and scanning efficiency to further increase its clinical applicability. Fourth, because V/Q SPECT-CT was not clinically available at our center, we could not correlate PREFUL-MRI parameters with the current reference standard for regional ventilation/perfusion imaging. Future multicenter work should directly compare these two techniques to determine diagnostic concordance and possible complementary roles. Fifth, HRCT quantification was performed in a commercial AI workstation; whether other AI platforms would produce identical fibrosis scores remains unknown, and future work should directly compare multiple algorithms or employ a phantom-based standard to assess cross-vendor reproducibility. In addition, it is particularly important to emphasize that PREFUL MRI could serve as a valuable adjunct to HRCT and pulmonary function test for the assessment of F-ILD, but it cannot replace HRCT and pulmonary function.

## Conclusions

PREFUL MRI provides a non-invasive, radiation-free method for quantitatively assessing lung perfusion and ventilation in F-ILD patients. It offers strong correlations with PFTs and fibrotic lesions identified on HRCT, positioning it as a valuable tool for monitoring disease severity. The independent predictive power of PREFUL MRI parameters, even after adjusting for demographic factors, underscores their potential as robust biomarkers for clinical decision-making and research. Future work should focus on validating these findings in larger, multi-center cohorts and exploring the longitudinal utility of PREFUL MRI in tracking disease progression and treatment response.

## ELECTRONIC SUPPLEMENTARY MATERIAL

Supplementary Material

## Data Availability

The datasets and material generated or analyzed during the study are available from the corresponding author on reasonable request.

## Abbreviations

ATS: American Thoracic Society
COPD: Chronic obstructive pulmonary disease
DLCO SB %pred: Percentage of predicted single breath diffusing capacity for carbon monoxide
DLCO/VA %pred: Percentage of predicted diffusing capacity for carbon monoxide divided by the alveolar volume
FEV1%pred: Percentage of predicted forced expiratory volume in one second
F-ILD: Fibrosing interstitial lung disease
FVC %pred: Percentage of predicted forced vital capacity
FVL: Flow volume loop
GGO: Ground-glass opacity
HRCT: High-resolution CT
ILD: Interstitial lung disease
IPF: Idiopathic pulmonary fibrosis
LDCT: Low-dose CT
Mean FVL correlation: Mean flow volume loop correlation
PFTs: Pulmonary function tests
PREFUL MRI: Phase-resolved functional lung magnetic resonance imaging
QDPexclusive: Percentage of areas with perfusion defects but without ventilation defects
SD: Standard deviation
TLC %pred: Percentage of predicted total lung capacity
VC %pred: Percentage of predicted vital capacity
VDPexclusive: Percentage of areas with ventilation defects but without perfusion defects
VQMdefect: Percentage of areas with concurrent perfusion and ventilation defects
VQMnon-defect: Percentage of areas without perfusion defects or ventilation defects
