# Supplementary material for: Phase-resolved functional lung magnetic resonance imaging for evaluation of lung perfusion and ventilation in fibrosing interstitial lung diseases
Source: Insights Imaging. 2026 Jun 4;17:151. doi: 10.1186/s13244-026-02321-5 (PMC13237287; doi:10.1186/s13244-026-02321-5)
Supplement: Supplementary file 1 — Supplementary Material [file 13244_2026_2321_MOESM1_ESM.pdf]

Phase-Resolved Functional Lung Magnetic Resonance Imaging for evaluation of lung perfusion and ventilation in Fibrosing Interstitial Lung Diseases

ELECTRONIC SUPPLEMENTARY MATERIAL

Insights Imaging (2026) Ni Y, Wang H, Liu A, et al.

Supplementary Table S1. Definitions of parameters

Parameter, units	Definitions
Mean perfusion, %	Perfusion was normalized in reference to a full-blood signal region, derived as follows: (1) determine the parenchyma cardiac cycle phase through histogram analysis of the signal in the lung parenchyma, calculated as the time point in the reconstructed cardiac cycle when most of the lung parenchyma voxels reach their maximal value; (2) locate the full-blood signal region for normalization as the maximal intensity projection value in the lungs that which may to reflect the aorta or other available large vessel; and (3) divide the map of the reconstructed cardiac cycle signal at the parenchyma phase by the maximal value in the normalization region.
Mean ventilation, %	Fractional ventilation of the whole respiratory cycle calculated as: $\frac{S_{Mid}}{S_{Insp}} - \frac{S_{Mid}}{S_{Exp}}$ with S the signal value at end-inspiration (*Insp*), end-expiration (*Exp*) and middle position (*Mid*)

VDP_{total}, %	Percentage of areas identified as ventilation defects in total
$VDP_{exclusive}$, %	Percentage of areas with ventilation defects but without perfusion defects
QDP_{change}	The difference between QDP and its cut-off value, with values greater than zero assigned as 1 and others as 0
QDP_{total}, %	Percentage of areas identified as perfusion defect in total
$QDP_{exclusive}$, %	Percentage of areas with perfusion defects but without ventilation defects
VDP_{change}	The difference between VDP and its cut-off value, with values greater than zero assigned as 1 and others as 0
VQM_{defect}, %	Percentage of areas with concurrent perfusion and ventilation defects
$VQM_{non\text{-}defect}$, %	Percentage of areas without perfusion defects or ventilation defects

Supplementary Table S2. Correlation analysis between PREFUL MRI related parameters and Pulmonary Function in F-ILD Patients.

Var1	Var2	Correlation（ρ）	*p*-value	FDR-adjusted *p*
Mean Perfusion (%)	***DLCO SB %pred***	***0.682***	***<0.001****	***0.001****
Mean Perfusion (%)	***DLCO/VA %pred***	***0.634***	***<0.001****	***0.003****
**Mean Perfusion (%)**	**FEV1**	**0.013**	**0.945**	**0.992**
**Mean Perfusion (%)**	**FEV1/FVC**	**0.264**	**0.158**	**0.802**
**Mean Perfusion (%)**	**FEV1/FVC %pred**	**0.249**	**0.184**	**0.759**
**Mean Perfusion (%)**	**FEV1 %pred**	**0.069**	**0.717**	**0.992**
**Mean Perfusion (%)**	**FVC**	**-0.059**	**0.757**	**0.992**
**Mean Perfusion (%)**	**FVC %pred**	**0.004**	**0.983**	**0.992**
**Mean Perfusion (%)**	**TLC-SB**	**-0.092**	**0.627**	**0.992**
**Mean Perfusion (%)**	**TLC-SB %pred**	**0.105**	**0.581**	**0.992**

**Mean Perfusion (%)**	**VC %pred**	**0.015**	**0.938**	**0.992**
**Mean Ventilation (%)**	**DLCO SB %pred**	**0.106**	**0.578**	**0.992**
**Mean Ventilation (%)**	**DLCO/VA %pred**	**0.019**	**0.921**	**0.992**
**Mean Ventilation (%)**	**FEV1**	**0.171**	**0.367**	**0.970**
**Mean Ventilation (%)**	**FEV1/FVC**	**-0.046**	**0.808**	**0.992**
**Mean Ventilation (%)**	**FEV1/FVC %pred**	**-0.035**	**0.854**	**0.992**
**Mean Ventilation (%)**	**FEV1 %pred**	**0.091**	**0.632**	**0.992**
**Mean Ventilation (%)**	**FVC**	**0.165**	**0.383**	**0.971**
**Mean Ventilation (%)**	**FVC %pred**	**0.145**	**0.446**	**0.992**
**Mean Ventilation (%)**	**TLC-SB**	**0.096**	**0.615**	**0.992**
**Mean Ventilation (%)**	**TLC-SB %pred**	**0.136**	**0.474**	**0.992**
**Mean Ventilation (%)**	**VC %pred**	**0.118**	**0.534**	**0.992**
$QDP_{exclusive}$ (%)	***DLCO SB %pred***	***-0.688***	***<0.001****	***0.002****
$QDP_{exclusive}$ (%)	***DLCO/VA %pred***	***-0.645***	***<0.001****	***0.003****

$QDP_{exclusive}$ (%)	FEV1	0.042	0.826	0.992
$QDP_{exclusive}$ (%)	FEV1/FVC	-0.263	0.160	0.755
$QDP_{exclusive}$ (%)	FEV1/FVC %pred	-0.249	0.184	0.809
$QDP_{exclusive}$ (%)	FEV1 %pred	-0.045	0.814	0.992
$QDP_{exclusive}$ (%)	FVC	0.110	0.562	0.992
$QDP_{exclusive}$ (%)	FVC %pred	-0.017	0.929	0.992
$QDP_{exclusive}$ (%)	TLC-SB	0.107	0.575	0.992
$QDP_{exclusive}$ (%)	TLC-SB %pred	-0.132	0.486	0.992
$QDP_{exclusive}$ (%)	VC %pred	-0.014	0.943	0.992
QDP_{change}	DLCO SB %pred	-0.315	0.090*	0.543
QDP_{change}	DLCO/VA %pred	-0.404	0.027	0.196
QDP_{change}	FEV1	0.019	0.920	0.995
QDP_{change}	FEV1/FVC	-0.212	0.261	0.862
QDP_{change}	FEV1/FVC %pred	-0.212	0.261	0.907

QDP_{change}	FEV1 %pred	0.199	0.292	0.875
QDP_{change}	FVC	0.032	0.866	0.995
QDP_{change}	FVC %pred	0.225	0.233	0.853
QDP_{change}	TLC-SB	0.032	0.866	0.995
QDP_{change}	TLC-SB %pred	0.083	0.661	0.992
QDP_{change}	VC %pred	0.225	0.233	0.903
$VDP_{exclusive}$ (%)	DLCO SB %pred	0.191	0.311	0.892
$VDP_{exclusive}$ (%)	DLCO/VA %pred	0.278	0.137	0.751
$VDP_{exclusive}$ (%)	FEV1	0.139	0.463	0.995
$VDP_{exclusive}$ (%)	FEV1/FVC	-0.428	0.018*	0.174
$VDP_{exclusive}$ (%)	FEV1/FVC %pred	-0.417	0.022*	0.180
$VDP_{exclusive}$ (%)	FEV1 %pred	-0.046	0.810	0.995
$VDP_{exclusive}$ (%)	FVC	0.202	0.285	0.897
$VDP_{exclusive}$ (%)	FVC %pred	-0.008	0.967	0.995

**$VDP_{exclusive}$ (%)**	**TLC-SB**	**0.319**	**0.085**	**0.563**
**$VDP_{exclusive}$ (%)**	**TLC-SB %pred**	**0.084**	**0.658**	**0.995**
**$VDP_{exclusive}$ (%)**	**VC %pred**	**0.015**	**0.938**	**0.995**
**VDP_{change}**	**DLCO SB %pred**	**0.068**	**0.721**	**0.995**
**VDP_{change}**	**DLCO/VA %pred**	**0.108**	**0.570**	**0.995**
**VDP_{change}**	**FEV1**	**-0.084**	**0.659**	**0.995**
**VDP_{change}**	**FEV1/FVC**	**-0.460**	**0.011***	**0.117**
**VDP_{change}**	**FEV1/FVC %pred**	**-0.460**	**0.011***	**0.140**
**VDP_{change}**	**FEV1 %pred**	**-0.092**	**0.629**	**0.995**
**VDP_{change}**	**FVC**	**0.004**	**0.983**	**0.983**
**VDP_{change}**	**FVC %pred**	**-0.028**	**0.883**	**0.995**
**VDP_{change}**	**TLC-SB**	**0.176**	**0.353**	**0.970**
**VDP_{change}**	**TLC-SB %pred**	**0.084**	**0.659**	**0.995**
**VDP_{change}**	**VC %pred**	**0.012**	**0.950**	**0.995**

Notes: $VDP_{exclusive}$: percentage of areas with ventilation defects but without perfusion defects; $QDP_{exclusive}$: percentage of areas with perfusion defects but without ventilation defects; QDP_{change} was defined as the difference between QDP and its cut-off value, with values greater than zero assigned as 1 and others as 0. Similarly, VDP_{change} was calculated as the difference between VDP and its cut-off value, also categorized as 1 if greater than zero and 0 otherwise; FVC %pred: percentage of predicted forced vital capacity; FEV1 %pred: percentage of predicted forced expiratory volume in one second; VC %pred: percentage of predicted vital capacity; TLC %pred: percentage of predicted total lung capacity; DLCO SB %pred: percentage of predicted single breath diffusing capacity for carbon monoxide; DLCO/VA %pred : percentage of predicted diffusing capacity for carbon monoxide divided by the alveolar volume. *Statistically significant, $p<0.05$.

Supplementary Table S3. Correlation Between PREFUL MRI related parameters and Lung Lesions on HRCT in F-ILD Patients.

Var1（PREFUL MRI）	Var2（Lung Lesions on HRCT）	Correlation（ρ）	*p*-value	FDR-adjusted *p*
Mean Perfusion (%)	The whole-lung percentage of GGO (%)	-0.169	0.371	0.614
Mean Perfusion (%)	The whole-lung percentage of emphysema (%)	-0.316	0.089	0.305
Mean Perfusion (%)	The whole-lung percentage of honeycombing (%)	-0.447	0.013*	0.125
Mean Perfusion (%)	The whole-lung percentage of consolidation (%)	0.352	0.057	0.228

**Mean Perfusion (%)**	**The whole-lung percentage of reticulation (%)**	**-0.404**	**0.027***	**0.162**
**Mean Perfusion (%)**	**The whole-lung percentage of non-fibrotic lesions (%)**	**-0.169**	**0.371**	**0.614**
**Mean Perfusion (%)**	**The whole-lung percentage of fibrotic lesions (%)**	**-0.481**	**0.007***	**0.112**
Mean Perfusion (%)	***Total interstitial lesion burden (%)***	***-0.600***	***<0.001****	***<0.001****
**Mean Ventilation (%)**	**The whole-lung percentage of GGO (%)**	**0.200**	**0.289**	**0.631**
**Mean Ventilation (%)**	**The whole-lung percentage of emphysema (%)**	**-0.249**	**0.185**	**0.493**
**Mean Ventilation (%)**	**The whole-lung percentage of honeycombing (%)**	**-0.229**	**0.225**	**0.540**
**Mean Ventilation (%)**	**The whole-lung percentage of consolidation (%)**	**0.037**	**0.847**	**0.924**

**Mean Ventilation (%)**	**The whole-lung percentage of reticulation (%)**	**0.071**	**0.710**	**0.852**
**Mean Ventilation (%)**	**The whole-lung percentage of non-fibrotic lesions (%)**	**0.200**	**0.289**	**0.631**
**Mean Ventilation (%)**	**The whole-lung percentage of fibrotic lesions (%)**	**-0.004**	**0.985**	**0.985**
**Mean Ventilation (%)**	**Total interstitial lesion burden (%)**	**-0.181**	**0.338**	**0.579**
**$QDP_{exclusive}$ (%)**	**The whole-lung percentage of GGO (%)**	**0.189**	**0.316**	**0.607**
**$QDP_{exclusive}$ (%)**	**The whole-lung percentage of emphysema (%)**	**0.379**	**0.039***	**0.208**
**$QDP_{exclusive}$ (%)**	**The whole-lung percentage of honeycombing (%)**	**0.461**	**0.010***	**0.120**
**$QDP_{exclusive}$ (%)**	**The whole-lung percentage of consolidation (%)**	**-0.310**	**0.095**	**0.304**

**$QDP_{exclusive}$ (%)**	**The whole-lung percentage of reticulation (%)**	**0.410**	**0.024***	**0.165**
**$QDP_{exclusive}$ (%)**	**The whole-lung percentage of non-fibrotic lesions (%)**	**0.189**	**0.316**	**0.607**
**$QDP_{exclusive}$ (%)**	**The whole-lung percentage of fibrotic lesions (%)**	**0.446**	**0.013***	**0.125**
$QDP_{exclusive}$ (%)	***Total interstitial lesion burden (%)***	***0.610***	***<0.001****	***<0.001****
**QDP_{change}**	**The whole-lung percentage of GGO (%)**	**0.052**	**0.783**	**0.917**
**QDP_{change}**	**The whole-lung percentage of emphysema (%)**	**0.230**	**0.221**	**0.558**
**QDP_{change}**	**The whole-lung percentage of honeycombing (%)**	**0.362**	**0.049***	**0.235**
**QDP_{change}**	**The whole-lung percentage of consolidation (%)**	**-0.188**	**0.319**	**0.567**

**QDP_{change}**	**The whole-lung percentage of reticulation (%)**	**0.296**	**0.112**	**0.316**
**QDP_{change}**	**The whole-lung percentage of non-fibrotic lesions (%)**	**0.052**	**0.783**	**0.917**
**QDP_{change}**	**The whole-lung percentage of fibrotic lesions (%)**	**0.302**	**0.105**	**0.315**
**QDP_{change}**	**Total interstitial lesion burden (%)**	**0.276**	**0.139**	**0.262**
**$VDP_{exclusive}$ (%)**	**The whole-lung percentage of GGO (%)**	**-0.082**	**0.668**	**0.844**
**$VDP_{exclusive}$ (%)**	**The whole-lung percentage of emphysema (%)**	**0.206**	**0.274**	**0.626**
**$VDP_{exclusive}$ (%)**	**The whole-lung percentage of honeycombing (%)**	**0.103**	**0.589**	**0.764**
**$VDP_{exclusive}$ (%)**	**The whole-lung percentage of consolidation (%)**	**0.116**	**0.540**	**0.720**

**$VDP_{exclusive}$ (%)**	**The whole-lung percentage of reticulation (%)**	**0.134**	**0.481**	**0.679**
**$VDP_{exclusive}$ (%)**	**The whole-lung percentage of non-fibrotic lesions (%)**	**-0.082**	**0.668**	**0.844**
**$VDP_{exclusive}$ (%)**	**The whole-lung percentage of fibrotic lesions (%)**	**0.124**	**0.515**	**0.706**
**$VDP_{exclusive}$ (%)**	**Total interstitial lesion burden (%)**	**0.005**	**0.979**	**1**
**VDP_{change}**	**The whole-lung percentage of GGO (%)**	**-0.145**	**0.446**	**0.669**
**VDP_{change}**	**The whole-lung percentage of emphysema (%)**	**0.363**	**0.049***	**0.235**
**VDP_{change}**	**The whole-lung percentage of honeycombing (%)**	**0.146**	**0.441**	**0.683**
**VDP_{change}**	**The whole-lung percentage of consolidation (%)**	**0.197**	**0.296**	**0.592**

**VDP_{change}**	**The whole-lung percentage of reticulation (%)**	**0.016**	**0.933**	**0.974**
**VDP_{change}**	**The whole-lung percentage of non-fibrotic lesions (%)**	**-0.145**	**0.446**	**0.669**
**VDP_{change}**	**The whole-lung percentage of fibrotic lesions (%)**	**0.028**	**0.883**	**0.942**
**VDP_{change}**	**Total interstitial lesion burden (%)**	**0.044**	**0.817**	**0.912**

Notes: Notes: $VDP_{exclusive}$: percentage of areas with ventilation defects but without perfusion defects; $QDP_{exclusive}$: percentage of areas with perfusion defects but without ventilation defects; QDP_{change} was defined as the difference between QDP and its cut-off value, with values greater than zero assigned as 1 and others as 0. Similarly, VDP_{change} was calculated as the difference between VDP and its cut-off value, also categorized as 1 if greater than zero and 0 otherwise; GGO: ground-glass opacity. *Statistically significant, $p<0.05$.

Supplementary Table S4. Correlation Between Lung Lesions on HRCT and Pulmonary Function Tests in F-ILD Patients

Var1 (Lung Lesions on HRCT)	Var2 (Pulmonary Function)	Correlation（ρ）	*p*-value	FDR-adjusted *p*
The whole-lung percentage of GGO (%)	DLCO SB %pred	-0.320	0.085	0.232
The whole-lung percentage of GGO (%)	DLCO/VA %pred	0.039	0.839	0.966
The whole-lung percentage of GGO (%)	FEV1	-0.101	0.594	0.824
The whole-lung percentage of GGO (%)	FEV1/FVC	0.091	0.632	0.845
The whole-lung percentage of GGO (%)	FEV1/FVC %pred	0.092	0.630	0.844
The whole-lung percentage of GGO (%)	FEV1 %pred	-0.442	0.015*	0.103
The whole-lung percentage of GGO (%)	FVC	-0.113	0.552	0.801
The whole-lung percentage of GGO (%)	FVC %pred	-0.405	0.026*	0.142
The whole-lung percentage of GGO (%)	TLC-SB	-0.033	0.864	0.974
The whole-lung percentage of GGO (%)	TLC-SB %pred	-0.210	0.266	0.558
The whole-lung percentage of GGO (%)	VC %pred	-0.430	0.018*	0.116

The whole-lung percentage of emphysema (%)	DLCO SB %pred	-0.394	0.031*	0.169
The whole-lung percentage of emphysema (%)	DLCO/VA %pred	-0.318	0.087	0.236
The whole-lung percentage of emphysema (%)	FEV1	-0.102	0.591	0.822
The whole-lung percentage of emphysema (%)	FEV1/FVC	-0.214	0.257	0.546
The whole-lung percentage of emphysema (%)	FEV1/FVC %pred	-0.198	0.293	0.590
The whole-lung percentage of emphysema (%)	FEV1 %pred	-0.241	0.200	0.482
The whole-lung percentage of emphysema (%)	FVC	-0.063	0.743	0.912

**The whole-lung percentage of emphysema (%)**	**FVC %pred**	**-0.201**	**0.288**	**0.584**
**The whole-lung percentage of emphysema (%)**	**TLC-SB**	**0.029**	**0.877**	**0.979**
**The whole-lung percentage of emphysema (%)**	**TLC-SB %pred**	**-0.168**	**0.374**	**0.683**
**The whole-lung percentage of emphysema (%)**	**VC %pred**	**-0.158**	**0.405**	**0.708**
The whole-lung percentage of honeycombing (%)	***DLCO SB %pred***	***-0.566***	***0.001****	***0.014****
**The whole-lung percentage of honeycombing (%)**	**DLCO/VA %pred**	**-0.422**	**0.020***	**0.125**
**The whole-lung percentage of honeycombing (%)**	**FEV1**	**-0.235**	**0.212**	**0.500**

The whole-lung percentage of honeycombing (%)	FEV1/FVC	-0.100	0.601	0.828
The whole-lung percentage of honeycombing (%)	FEV1/FVC %pred	-0.078	0.683	0.879
The whole-lung percentage of honeycombing (%)	FEV1 %pred	-0.174	0.358	0.669
The whole-lung percentage of honeycombing (%)	FVC	-0.209	0.267	0.560
The whole-lung percentage of honeycombing (%)	FVC %pred	-0.199	0.291	0.588
The whole-lung percentage of honeycombing (%)	TLC-SB	-0.158	0.403	0.708
The whole-lung percentage of honeycombing (%)	TLC-SB %pred	-0.313	0.092	0.245

**The whole-lung percentage of honeycombing (%)**	**VC %pred**	**-0.174**	**0.357**	**0.669**
**The whole-lung percentage of consolidation (%)**	**DLCO SB %pred**	**-0.114**	**0.550**	**0.800**
**The whole-lung percentage of consolidation (%)**	**DLCO/VA %pred**	**0.273**	**0.144**	**0.342**
The whole-lung percentage of consolidation (%)	***FEV1***	***-0.578***	***0.001****	***0.014****
**The whole-lung percentage of consolidation (%)**	**FEV1/FVC**	**0.139**	**0.462**	**0.750**
**The whole-lung percentage of consolidation (%)**	**FEV1/FVC %pred**	**0.135**	**0.478**	**0.761**
The whole-lung percentage of consolidation (%)	***FEV1 %pred***	***-0.525***	***0.003****	***0.028****

The whole-lung percentage of consolidation (%)	*FVC*	*-0.576*	*0.001**	*0.014**
The whole-lung percentage of consolidation (%)	*FVC %pred*	*-0.515*	*0.004**	*0.033**
The whole-lung percentage of consolidation (%)	TLC-SB	-0.458	0.011*	0.086
The whole-lung percentage of consolidation (%)	TLC-SB %pred	-0.393	0.032*	0.163
The whole-lung percentage of consolidation (%)	*VC %pred*	*-0.495*	*0.005**	*0.039**
The whole-lung percentage of reticulation (%)	*DLCO SB %pred*	*-0.651*	*<0.001**	*<0.001**
The whole-lung percentage of reticulation (%)	DLCO/VA %pred	-0.280	0.134	0.323

**The whole-lung percentage of reticulation (%)**	**FEV1**	**-0.276**	**0.139**	**0.331**
**The whole-lung percentage of reticulation (%)**	**FEV1/FVC**	**0.147**	**0.437**	**0.731**
**The whole-lung percentage of reticulation (%)**	**FEV1/FVC %pred**	**0.170**	**0.370**	**0.677**
**The whole-lung percentage of reticulation (%)**	**FEV1 %pred**	**-0.399**	**0.029***	**0.154**
**The whole-lung percentage of reticulation (%)**	**FVC**	**-0.314**	**0.091**	**0.243**
The whole-lung percentage of reticulation (%)	***FVC %pred***	***-0.480***	***0.007****	***0.048****
**The whole-lung percentage of reticulation (%)**	**TLC-SB**	**-0.275**	**0.141**	**0.337**

The whole-lung percentage of reticulation (%)	*TLC-SB %pred*	*-0.527*	*0.003**	*0.028**
The whole-lung percentage of reticulation (%)	VC %pred	-0.470	0.009*	0.062
The whole-lung percentage of non-fibrotic lesions (%)	DLCO SB %pred	-0.320	0.085	0.232
The whole-lung percentage of non-fibrotic lesions (%)	DLCO/VA %pred	0.039	0.839	0.966
The whole-lung percentage of non-fibrotic lesions (%)	FEV1	-0.092	0.629	0.843
The whole-lung percentage of non-fibrotic lesions (%)	FEV1/FVC	0.206	0.274	0.566
The whole-lung percentage of non-fibrotic lesions (%)	FEV1/FVC %pred	0.092	0.630	0.844

**The whole-lung percentage of non-fibrotic lesions (%)**	**FEV1 %pred**	**-0.429**	**0.018***	**0.116**
**The whole-lung percentage of non-fibrotic lesions (%)**	**FVC**	**-0.125**	**0.509**	**0.772**
**The whole-lung percentage of non-fibrotic lesions (%)**	**FVC %pred**	**-0.405**	**0.026***	**0.142**
**The whole-lung percentage of non-fibrotic lesions (%)**	**TLC-SB**	**-0.033**	**0.864**	**0.974**
**The whole-lung percentage of non-fibrotic lesions (%)**	**TLC-SB %pred**	**-0.219**	**0.245**	**0.531**
**The whole-lung percentage of non-fibrotic lesions (%)**	**VC %pred**	**-0.430**	**0.018***	**0.116**
The whole-lung percentage of fibrotic lesions (%)	***DLCO SB %pred***	***-0.684***	***<0.001****	***<0.001****

The whole-lung percentage of fibrotic lesions (%)	DLCO/VA %pred	-0.334	0.071	0.201
The whole-lung percentage of fibrotic lesions (%)	FEV1	-0.306	0.100	0.259
The whole-lung percentage of fibrotic lesions (%)	FEV1/FVC	0.075	0.694	0.885
The whole-lung percentage of fibrotic lesions (%)	FEV1/FVC %pred	0.093	0.625	0.841
The whole-lung percentage of fibrotic lesions (%)	FEV1 %pred	-0.365	0.047*	0.209
The whole-lung percentage of fibrotic lesions (%)	FVC	-0.307	0.099	0.257
The whole-lung percentage of fibrotic lesions (%)	FVC %pred	-0.417	0.022*	0.132

The whole-lung percentage of fibrotic lesions (%)	TLC-SB	-0.263	0.160	0.369
The whole-lung percentage of fibrotic lesions (%)	*TLC-SB %pred*	*-0.494*	*0.006**	*0.043**
The whole-lung percentage of fibrotic lesions (%)	VC %pred	-0.409	0.025*	0.139
Total interstitial lesion burden (%)	*DLCO SB %pred*	*-0.801*	*<0.001**	*<0.001**
Total interstitial lesion burden (%)	DLCO/VA %pred	-0.340	0.066	0.189
Total interstitial lesion burden (%)	FEV1	-0.414	0.023*	0.135
Total interstitial lesion burden (%)	FEV1/FVC	0.052	0.784	0.944
Total interstitial lesion burden (%)	FEV1/FVC %pred	0.062	0.746	0.914
Total interstitial lesion burden (%)	*FEV1 %pred*	*-0.531*	*0.003**	*0.028**
Total interstitial lesion burden (%)	FVC	-0.391	0.033*	0.166
Total interstitial lesion burden (%)	*FVC %pred*	*-0.540*	*0.002**	*0.022**

**Total interstitial lesion burden (%)**	**TLC-SB**	**-0.338**	**0.067**	**0.192**
Total interstitial lesion burden (%)	***TLC-SB %pred***	***-0.578***	***0.001****	***0.014****
Total interstitial lesion burden (%)	***VC %pred***	***-0.522***	***0.003****	***0.028****

Notes: FVC %pred: percentage of predicted forced vital capacity; FEV1 %pred: percentage of predicted forced expiratory volume in one second; VC %pred: percentage of predicted vital capacity; TLC %pred: percentage of predicted total lung capacity; DLCO SB %pred: percentage of predicted single breath diffusing capacity for carbon monoxide; DLCO/VA %pred : percentage of predicted diffusing capacity for carbon monoxide divided by the alveolar volume; GGO: ground-glass opacity. *Statistically significant, $p<0.05$.
